# Renewable energy forecasting using optimized quantum temporal model based on Ninja optimization algorithm

**DOI:** 10.1038/s41598-025-97109-w

**Published:** 2025-04-27

**Authors:** Mona Ahmed Yassen, El-Sayed M. El-kenawy, Mohamed Gamal Abdel-Fattah, Islam Ismael, Hossam El.Deen Salah Mostafa

**Affiliations:** 1 Department of Electronics and Communications Engineering, Faculty of Engineering, 35516 Mansoura, Egypt; 2 Department of Communications and Electronics, Delta Higher Institute of Engineering and Technology, 35111 Mansoura, Egypt

**Keywords:** Renewable energy, Machine learning, Wind, XAI, LSTM, GRU, Energy science and technology, Machine learning

## Abstract

Artificial intelligence allows improvements in renewable energy systems by increasing efficiency while enhancing reliability and reducing costs. Renewable energy forecasting receives substantial improvement by applying deep learning methods as one of its promising approaches. The research utilizes QTM with NiOA optimization for achieving maximum forecasting performance. NiOA functions through critical optimization processes when enhancing deep learning models with high accuracy for large complex datasets by selecting the most appropriate features. Fundamental data preparation steps, including normalization scaling, and gap handling, play a vital role before using input data for reliable renewable energy forecasting operations. Using the Ninja binary optimization engine produces superior results than all tested binary algorithms, including SBO, bSCA, bFA, bGA, bFEP, bGSA, bDE, bTSH and bBA, resulting in enhanced classification accuracy. The superior capability of bNinja to choose optimal features establishes its usefulness for renewable energy forecasting applications. Experimental implementation revealed that incorporating the Ninja Optimization Algorithm with the QTM model delivered the best R^2^ performance at 95.15% with an exceptional RMSE value of 0.00003, thus establishing its ability to optimize renewable energy forecasting accuracy.

## Introduction

Energy is called civilization’s essence and a part of people’s life. The availability of sustainable and renewable power sources is one of the critical factors in the present civilization’s success. However, the rapid industrialization and expansion of world civilization threaten the world’s reformed fossil fuel reserves and our environment. To mitigate these threats, sustainable development measures and standards must be incorporated into technical processes, products, and projects to prevent the exhaustion of global resources and reduce environmental pollution^1^. This urgency has led to an increased focus on creating renewable energy resources. Projections indicate that renewable energy capacity through renewable technologies like wind and solar will expand significantly in the coming years^2^. Using conventional power sources, such as fossil fuels, remains controversial due to natural resource depletion and climate change^3,4^. In contrast, wind and solar energy have emerged as green alternatives with the potential to reduce regional and global emissions while playing a central role in ecosystem conservation. As the detrimental effects of fossil fuel use become increasingly evident, the transition to renewable energy has gained momentum^5^. However, wind and solar energy present intrinsic issues related to the random nature of wind and solar power, making the balance of power system and system operations and control even more complex. For instance, depending on time, the wind has low reliability, compromised system reliability, and high integration costs, and wind generation is sometimes curtailed^6^. Addressing these challenges requires advanced predictive analysis tools and machine learning algorithms to improve wind power technology predictions. Enhanced forecasting accuracy will enable better integration of renewable energy into the grid, reducing interchange costs and improving system stability^7^. Thus, reaching global carbon neutrality is transferable by the complementary development of wind and solar energy industries. This approach helps ensure the reduction or total elimination of carbon dioxide in the atmosphere through technological development. According to the IEA, wind energy is projected to become the largest source of renewable electricity by 2050, with its share increasing by 35%^8^. Despite disruptions caused by the COVID-19 pandemic, wind power capacity has grown steadily, as highlighted by the Global Wind Energy Council (GWEC), with cumulative installations reaching 95.3 GW in 2020, 93.6 GW in 2021, and 77.6 GW in 2022. Such an increase can be attributed to the increasing demand for renewable energy globally, placing wind among the most sought green energy sources following hydropower^9^. Likewise, Solar energy is also a renewable resource that has easy availability, less cost in electricity bills, low running expenses and constant innovation.^10^. However, its adoption faces obstacles, including high equipment costs, weather dependency, complex storage systems, and significant space requirements^11^. Despite these challenges, photovoltaic (PV) technology has become a pivotal renewable energy application, with the Earth receiving an average solar radiation of approximately 1361 W/m^2^^12^. Investments in solar energy technology continue to grow, enhancing its potential^13^. Recent innovations, such as thin-film technologies and multifunctional photovoltaic cells, replace traditional crystalline silicon cells, driving solar power’s rapid expansion^14^. These advancements improve energy trading and management within power grids by reducing mismatches between supply and demand, thus lowering energy delivery costs and minimizing reliance on control energy^15^. Wind and solar energy are often complementary due to their shared role in meeting global energy demand. The Renewable Energy Policy Network forecasts that solar photovoltaic energy will achieve a capacity exceeding 8,000 GW by 2050. Active and passive solar energy systems increase flexibility by directly or indirectly utilizing photovoltaic energy or generating electricity from solar radiation. Despite challenges such as the limited availability of silicon-boron semiconductor chips and the intermittent nature of sunlight, technological advancements and grid management solidify solar energy’s place in a balanced renewable energy system. The inherent variability of renewable energy sources necessitates stochastic forecasting to improve energy storage and dispatch control systems. In other words, forecasting helps to improve explicit control over the grid by guaranteeing resource availability, veracity, and reliability. In WPFM methodologies, we have a deterministic prognosis, where one value is given for a time of day, and a probabilistic prognosis, which measures the risk of opting for one decision. Renewable energy systems (RES) are preferred over thermal generation systems due to their reduced greenhouse gas emissions, minimal climatic impact, and lower production and maintenance costs^16,17^. However, challenges such as variability and inefficiencies in estimation and optimization hinder their seamless integration into national grids^18,19^. Researchers are leveraging artificial intelligence (AI) to address these issues, predict variability, perform maximum power point tracking, and optimize hybrid RES systems. AI applications inspire optimism for the successful integration of renewable energy^20,21^. For example, Liu et al.^22^ combined the Light Gradient Boosting Machine (LGB) with Gaussian Process Regression (GPR) for precise deterministic and probabilistic wind speed predictions. Zhang et al.^23^ employed quantile regression to evaluate prediction uncertainties across various confidence intervals. Physical models like Numerical Weather Prediction (NWP)^24,25^ and Weather Research Forecasting (WRF) integrate meteorological and terrain data for wind speed calculations. Meanwhile, AI-based models, including Artificial Neural Networks (ANN), Support Vector Machines (SVM)^26^, and Ensemble Learning (EL) models such as Random Forest (RF) and Extreme Gradient Boosting (XGBoost)^27^, excel in capturing nonlinear patterns, making them highly effective for wind and solar power forecasting. Deep learning (DL) models^28^, such as Recurrent Neural Networks (RNN), Long Short-Term Memory (LSTM), and Convolutional Neural Networks (CNN), enhance prediction accuracy by extracting features from large datasets. For instance, Huang et al.^29^ incorporated KNN, RNN, LSTM, SVR, and RFM into a blended model to improve forecasting precision. Such advancements affirm the potential of AI in renewable energy forecasting. Beyond forecasting, AI also contributes to maximum power point tracking and hybrid system control, enabling the efficient integration of wind and solar energy into electricity systems. This reduces dependency on fossil fuels and fosters the transition to cleaner energy systems. The following stages of AI development are expected to resolve challenges related to renewable energy integration, making it widely available and stable to benefit the Earth. The key contributions of this study are as follows:

- This paper combines Ninja with Quantum Temporal Memory (QTM) networks to optimize hyperparameter tuning, thereby enhancing the model’s ability to predict renewable energy.
- A binary optimization approach is applied to select features from the dataset used in testing.
- To enhance the accuracy of the tested dataset’s classification, a weighted optimal deep learning model has been built. This model is based on the proposed Ninja technique.
- Several models were evaluated, including Convolution Neural Networks (CNN), Long Short-Term Memory (LSTM), Recurrent Neural Networks (RNN), Hybrid Encoder-Decoder Attention Models (HEDAMs), and Quantum Temporal Memory (QTM) demonstrating superior efficacy and delivering the most accurate predictions. Subsequently, Ninja was deployed to fine-tune the QTM model’s hyperparameters.
- A comparative study of the Ninja + QTM algorithm with other state-of-the-art algorithms was performed using the same dataset used in this paper.

The structure of this paper is as follows: Section “Related works” provides an overview of relevant state-of-the-art literature. Section “Materials and methods” provides a description of the data used and comprehensive review of the performance of the deep learning methods and materials utilized. Section “Proposed methodology” elaborates on the proposed approach. The results recorded from the conducted experiments are discussed in Section “Experimental results”. Finally, Section “Conclusion” presents the conclusions and future directions.

## Related works

The development of artificial intelligence models uses optimization algorithms with data preprocessing techniques to improve forecasting accuracy, especially in solar radiation and wind energy prediction^30^. The advancement of analytical tools that combine deep learning and metaheuristic algorithms assists in handling complex renewable energy forecasting operations. Power grids incorporating renewable sources depend on correct and immediate power predictions for managing their energy supply effectively while maintaining sustainable stability. The research findings demonstrate the importance of improving analytical methods with optimization techniques when dealing with forecasting challenges in renewable energy systems. For instance, Rafati et al.^31^ developed an MLP-NN model for daily solar power forecasting in the Netherlands using data preprocessing techniques such as RreliefF and Min-Max. These techniques improved performance accuracy based on MAE, MRE, and RMSE. However, results suggested that incorporating an additional optimization algorithm could enhance the search for the global minimum of the model. Regarding wind energy, the LSTM model was implemented in China for monthly wind power forecasting using CESM data, where results indicated that improving data preprocessing and incorporating optimization techniques could enhance forecasting capability^32^. Additionally, a Dual-Stage Attention LSTM model was applied in North China with Variational Mode Decomposition (VMD) for daily wind power forecasting, outperforming XGBoost, MLP, LSTM, CNN-LSTM, TPA-LSTM, and Transformer based on MAE, RMSE, and R^2^. This suggests that data decomposition and metaheuristic algorithms could significantly improve performance^33^. Similarly, López et al.^34^ proposed an LSTM model for wind speed forecasting, utilizing correlation matrix, normalization, structuring, and conversion techniques. It outperformed Persistence, MA, ARMA, NARX, NAR, and NIO in terms of R, MSE, and MAPE but still required further optimization to address the challenges of premature convergence. Researchers have focused on integrating metaheuristic optimization algorithms with AI models to address these limitations to enhance solar radiation and wind energy forecasting. For instance, Suo et al.^35^ demonstrated that optimizing BiGRU parameters using the improved Chaos Optimization Algorithm (IChOA) enhances forecasting performance. At the same time, Wei et al.^36^ optimized Deep Belief Networks (DBN) using the Self-adaptive Sparrow Optimization (SSO) algorithm to improve wind prediction capabilities. Similarly, Zhu et al.^37^ advanced wind power interval prediction by employing the Multi-Objective Evolutionary Chaos Optimization (MOECO) algorithm, outperforming traditional multi-objective optimization methods. Building upon these optimization techniques, Wu et al.^38^ applied adaptive differential evolution (JADE) to optimize the Temporal Fusion Transformer (TFT) model, ensuring stable and reliable Wind Speed Prediction (WSP) fusion. Likewise, Chen et al.^39^ used the Multi-Objective Slime Mould Algorithm (MOSMA) to fine-tune a DAE-GRU stacking model, achieving high accuracy and minimal bias. Wang et al.^40^ extended these improvements by adapting the Chaos-Enhanced Sparrow Search Optimization Algorithm (CSSOA) to LSTM networks, incorporating chaos sequences and a Gaussian mutation strategy to enhance wind power forecasting. Additionally, Ewees et al.^41^ demonstrated that the Harris Hawks Optimization (HBO) technique surpasses traditional metaheuristic methods like Genetic Algorithms (GA) in optimizing LSTM models. Despite these advancements, optimization techniques come with inherent limitations. Methods like HBO can suffer from premature convergence and overshooting errors, while Particle Swarm Optimization (PSO) excels in continuous optimization but struggles with local optima. The Grey Wolf Optimizer (GWO) is more efficient in global search tasks, whereas the Cuckoo algorithm demands extensive parameter tuning. Though more stable, multi-objective approaches such as MOCSO and MOECO face scalability challenges when applied to large datasets. Hossain et al.^42^ proposed a hybrid deep learning model that combines convolutional layers, gated recurrent unit (GRU) layers, and a fully connected neural network for very short-term predictions of wind power generation and achieved a significant improvement in accuracy for 5-minute interval predictions. An attention-based Bi-directional LSTM (BiLSTM) model was adopted for a farm in northern Kuwait, effectively capturing fluctuations in SE under varied conditions and achieving superior metrics like MSE, R-squared, NRMSE, and MAPE^43^. For large-scale datasets like the grid open dataset, a CNN-LSTM-Transformer model was designed to improve solar irradiance predictions while maintaining computational efficiency^44^. In Algeria, the BDSCA-LSTM model proved effective for estimating direct normal irradiance (DNI) in arid regions, demonstrating its applicability to climate-specific conditions with improved accuracy^45^. Similarly, DM Teferra et al.^46^ employed a hybrid fuzzy-PSO prediction model, integrating an error correction factor to enhance forecast accuracy for solar and wind power (data collected from Ethiopia’s National Metrology Agency). Again, utilizing the National Solar Radiation Database (NSRDB) dataset and ensemble Empirical Mode Decomposition (EEMD), A. Gupta and K. Gupta et al.^47^ proposed a hybrid approach named “EEMD-GA-LSTM,” which addressed the nonlinear characteristics of solar data, offering robust predictions with improved error metrics. Finally, the TNBR dataset utilized BPNN-PSO models, efficiently handling the nonlinear nature of solar irradiance data and providing accurate and precise predictions. These studies underscore the potential of hybrid and DL-based techniques in tackling the inherent complexities of solar radiation forecasting^48^. Moreover, recent research has focused on spatial and temporal correlations in wind-power forecasting. Wu et al.^49^ developed a spatiotemporal correlation model based on convolutional neural networks-long short memory (CNN-LSTM), outperforming single CNN and LSTM models. Neshat et al.^50^ developed a hybrid model combining quaternion convolutional neural networks with bidirectional LSTMs and adaptive variational mode decomposition methods, delivering outstanding results for short-term and long-term predictions. The research by Diaa Salman et al.^51^ analyzed solar power forecasting using a combination of CNN and LSTM and Transformer models, which delivered enhanced accuracy levels compared to singular models. The researchers emphasized that choosing model structures and optimization techniques improves predictive reliability. Bashir et al.^52^ developed two innovative hybrid CNN-ABiLSTM and CNN-Transformer-MLP forecasting models, which function for solar and wind power prediction. The unified approach delivered better forecasting results than the separate models by combining CNN short-term dependency modules alongside ABiLSTM/Transformer-MLP components for long-term trend analysis. Zhang et al.^53^ created an attention-based deep learning model using BiTCN and BiGRU with multi-head self-attention that achieved better wind power prediction accuracy by applying PCC framework for feature selection and CEEMDAN-based data decomposition. The research by Liu et al.^54^ presented a parallel architecture that united TCN and LSTM through a tensor concatenate module for improved computational efficiency. The combination of Savitzky-Golay (SG) filtering with their model structure reduced computational requirements without compromising prediction precision according to Diebold-Mariano testing and various evaluation metrics. Barjasteh et al.^55^ created a new short-term wind power forecasting system through the combination of Discrete Wavelet Transform (DWT) with recurrent neural network (RNN) models which included Bidirectional Long Short-Term Memory (BiLSTM) and Bidirectional Gated Recurrent Unit (BiGRU). The DWT decomposition separated wind speed traces into low-frequency portions and high-frequency components. The investigation findings demonstrate hybrid models serve as solutions to complex issues within renewable energy forecasting methods. Long-term dependency capture stands as a transformer model strength. In contrast, CNN models demonstrate enhanced feature extraction capabilities and computational efficiency, according to Bashir et al. and Salman et al., as well as Liu et al. and Zhang et al. New advancements demonstrate the persistent development of deep learning approaches for renewable energy prediction, leading to better and faster forecasting models. This paper presents a novel approach to renewable energy forecasting in desert areas to address these challenges the research aimed to construct an optimization method for parameter adjustment of the QTM system. Through NiOA decision makers achieve balance by avoiding local optimum traps while enhancing their chances to find global optimum solutions. The predictive accuracy and error reduction can be achieved through dynamic model parameter adjustments enabled by NiOA. These optimization problems benefit from NiOA and similar metaheuristic algorithms because they solve non-convex problems and multiple local optima, which prevent standard deterministic algorithms from discovering the best solution. NiOA improves QTM for renewable energy forecasting by incorporating multi-modal functions combined with adaptive search regulation. The prediction model included environmental data to detect complex wind-power production relationships between natural conditions and climate variables.

## Materials and methods

This section explains the following parts of the study: the deep learning models used to do the research, the dataset used to anticipate renewable energy, and the meta-heuristic optimization approaches used to optimize the models used in the studies.

### Dataset description

**Fig. 1:**
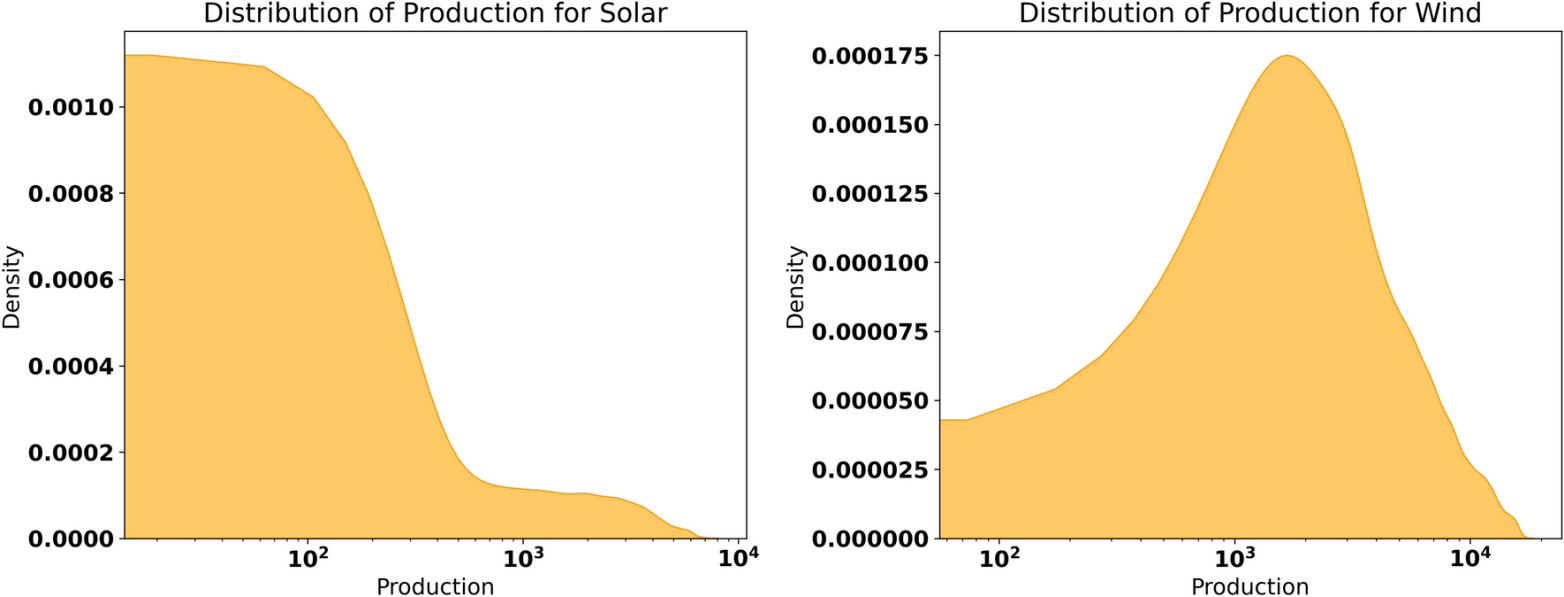
The production distribution for solar and wind energy.

**Fig. 2:**
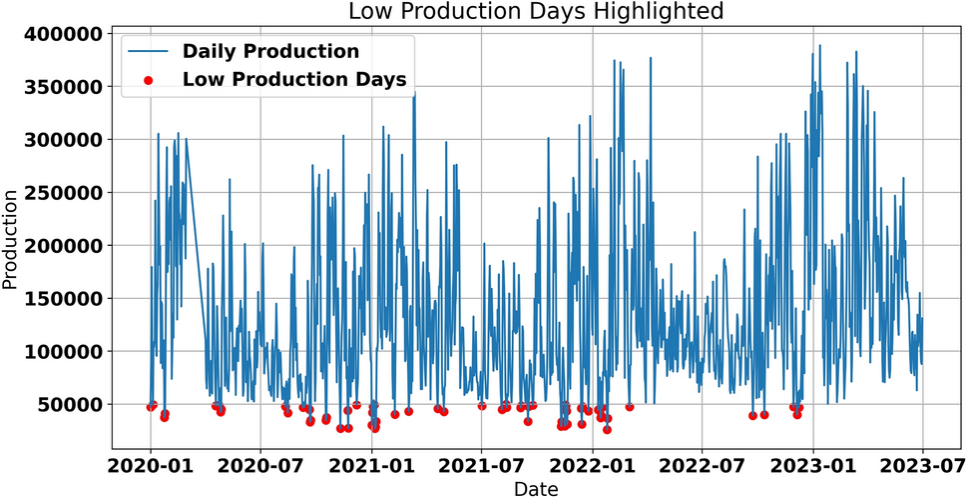
Daily renewable energy production with low-production days highlighted.

This dataset consists of wind and solar energy production (in MW) records hourly for the French grid since 2020. It provides extensive information regarding both production quantities and temporal characteristics. The Date and Hour range highlights the duration covered in the data collection, while the Date and Hour columns specify the exact timestamps for each energy generation record. Temporal granularity is further enriched by the StartHour and EndHour features, which indicate the precise start and end times of each recording session. The dataset includes the dayName and monthName features to analyze weekly and monthly patterns. Additionally, the day of year and Date features are critical for studying daily and seasonal trends in renewable energy production. The geographical location of power plants can also be compared. Still, the most important attribute here is the Source where a clear differentiation between wind and solar energy production is made. The production feature expressed in megawatt-hour (MWh) looks into the quantitative aspect of the energy produced from which performance comparison, randomness, and prediction can be derived. Figure 1 compares solar and wind energy production density. Consequently, the two sub-areas of renewable energy are still distinct in their characteristics. Therefore, the solar energy distribution shaping is positively biased, with most production values around average levels. In contrast, a few peak production values are experienced mid-day or where there is a clear sky. This variability reflects the influence of weather patterns and the sun’s position, which vary across seasons and times of the day. On the other hand, the wind energy distribution appears more uniform, with most production values concentrated in the middle range. This suggests that wind energy is less volatile than solar energy, as wind speed and climate conditions influence its production more consistently. Wind energy also demonstrates higher production capacity and better distribution than solar energy. Figure 2 is related to daily product production trend, with small red circles indicating low production days. The blue line scale shows daily production values for the year 2020 up to the date of mid-June 2023. Analyzing this graph, one can point out cyclic changes in renewable energy generation that correspond to the nature of wind and solar energy. Low-production days, although infrequent, are consistently observed throughout the dataset. These events may be occasioned by unsuitable climate conditions, including low winds or weak sunlight at times of the day or year when clouds obscure the sun or winter is setting in. Knowledge of such patterns is crucial to defining measures that can help lessen variability consequent on grid instability and energy supply unreliability.

### Metaheuristic optimization

**Fig. 3:**
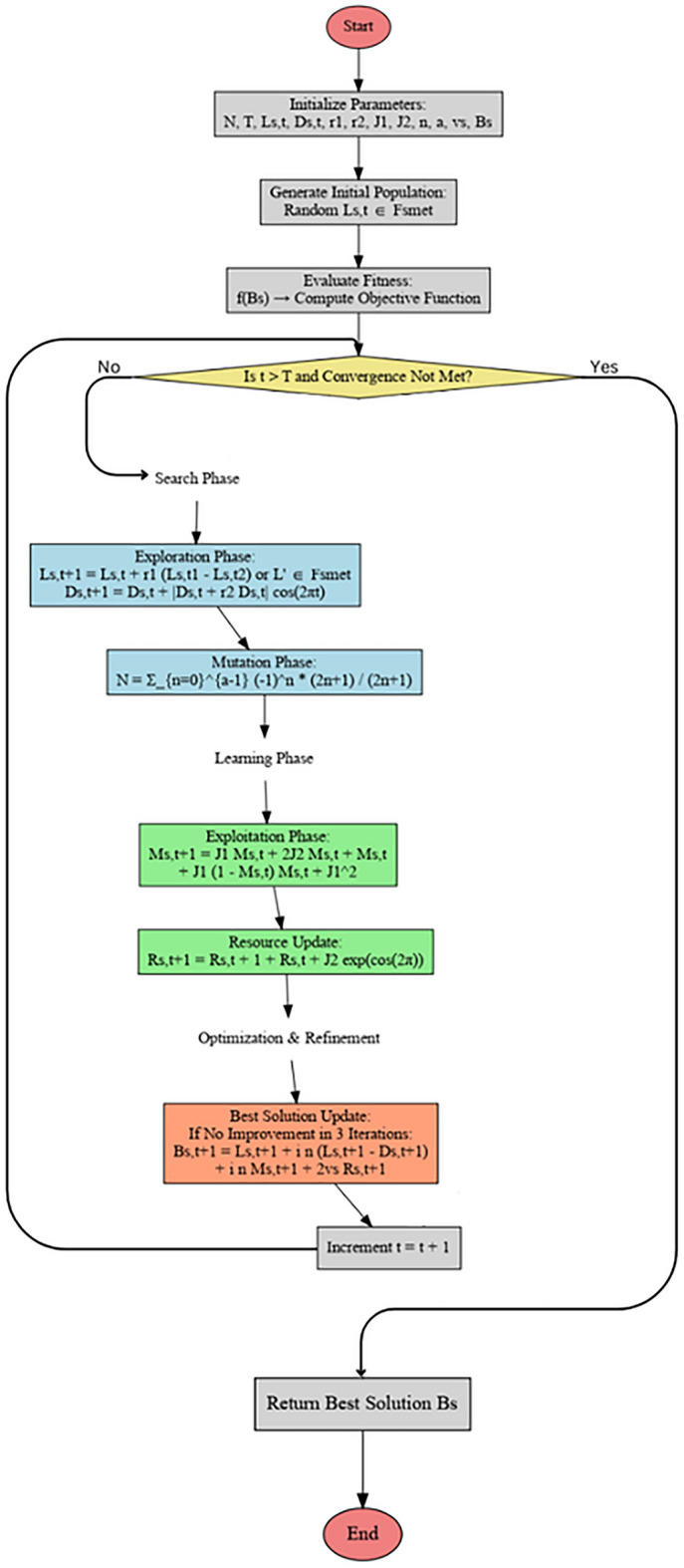
Flowchart of the proposed Ninja Optimization Algorithm.

The proposed optimization algorithm is shown in the flowchart depicted in Fig. 3.

The Ninja Optimizer Algorithm (NiOA) is the primary optimization technique in this study. NiOA integrates exploration and exploitation phases for solution search processes to evade local optima, which allows new solutions throughout extensive selections and better solutions from specific solution space areas. Maintaining a correct balance between exploration and exploitation phases enables the avoidance of local optima, which appear optimal at first but turn out suboptimal at the global level. Several sample metaheuristic programs were employed for testing to evaluate the performance levels of NiOA. The financial nature of the problem requires mathematical guidance for its optimizer to explore solution space successfully; the SCA trigonometric function approach helps the optimizer locate new solution areas and improve existing visited areas. New strategies to reach optimal solutions exist within the Harris Hawks Optimization Algorithm (HHO) because this algorithm applies the hunting methods of Harris Hawks. This behavior replicates the natural process, demonstrating how specific animals succeed better at catching prey under certain environmental conditions that help protect the hawk’s survival. The JAYA algorithm is a simple yet effective optimization method that targets improving solutions around efficient candidates and their reverse movement from inefficient solutions. Such an approach represents the optimal choice because it eliminates complex mathematical computations, which results in both efficiency and low computational cost. These optimization approaches exhibit different operational principles when they work to enhance machine learning algorithms used in the study^56^.

### Deep learning model for predicting renewable energy

This section introduced four deep algorithms: HEDAMs, LSTM, CNN and RNN.

#### Hybrid encoder–decoder attention models (HEDAMs)

**Fig. 4:**
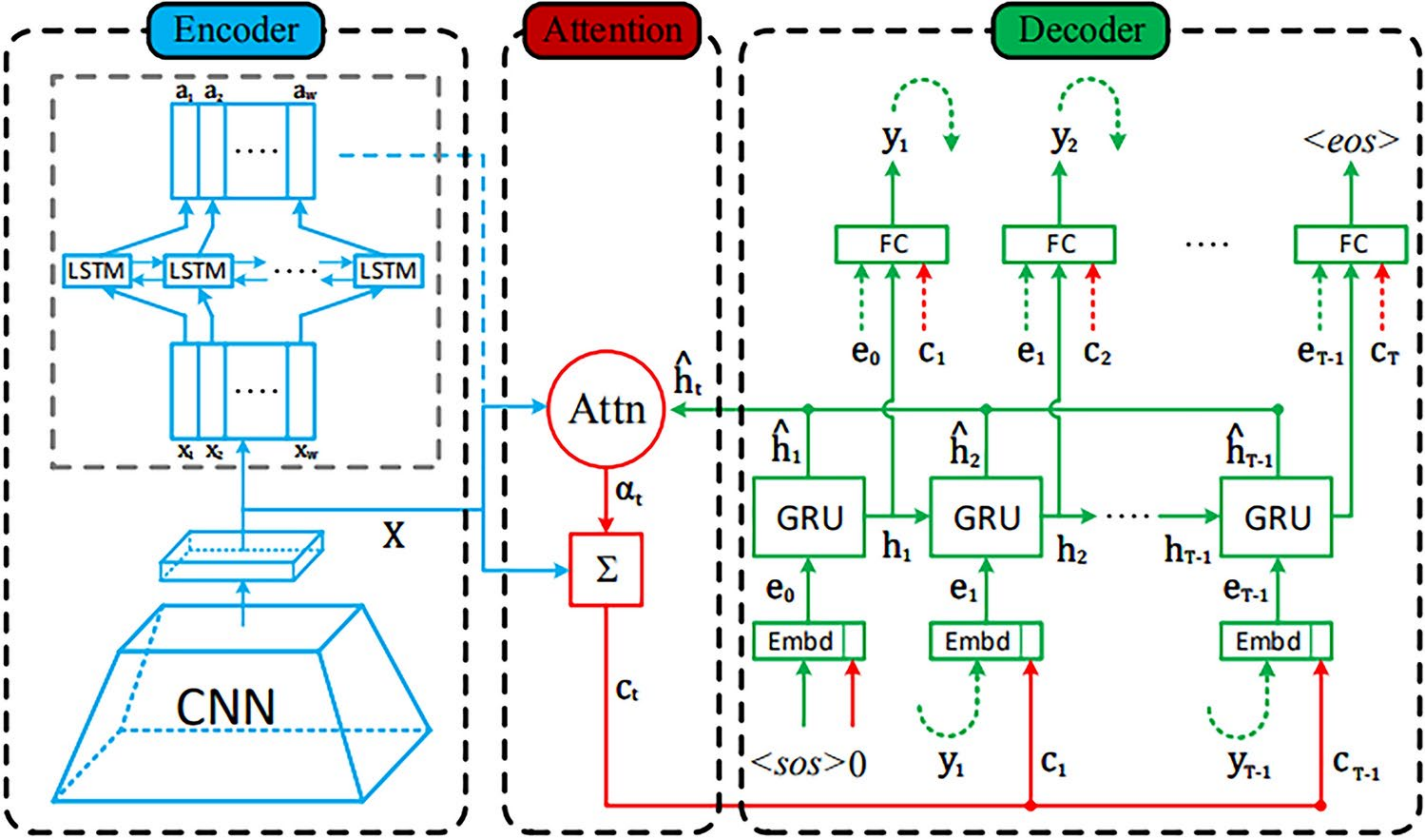
An example of an encoder-decoder structure based on attention.

Figure 4 below illustrates the general structure of an attention-based encoder-decoder approach. Consisting of three modules: decoder, attention and encoder. As for each of the above-stated components, a different model could be used. The whole information sequence is mapped into a fixed length of semantics vector by the encoder, which is a disadvantage of the basic encoder decoder idea. This approach has two shortcomings: First, the semantic vector can not represent the whole information sequence; second, the information behind the phrase will cover up the information in front of the sentence. These phenomena worsen when the input sequence size is more significant. As such, having enough information for the input sequence at the beginning of the decoding process becomes very challenging, which always results in poor performance. It does not function this way as the attention mechanism model does effectively if you ask me^57^.

#### Long short term memory (LSTM)

The use of LSTM Networks was also motivated by their well-established capacity to manage long-term dependencies on sequential data in earlier research. LSTMs use specialized memory blocks called cells, where we have not three but four types of controls on data flow: input, output and forget gates. Moving through a cell is the information, and those gates are its caretakers. Represented by an equation, the forget gate plays a crucial role in the LSTM network. It effectively identifies these data in the previous cell state that are no longer useful for computing and eliminates them, thus improving the network performance. The definition of the forget gate is:

$$x_t = \sigma (Q_x \cdot [h_{t-1}, y_t] + b_x)$$

where $\sigma$ is the sigmoid function, $Q_x$ is the weight matrix, and $b_x$ is the bias vector. The input gate, a key component, is responsible for updating the cell state with new information, as depicted by the equations:

$$\begin{aligned} i_t= & \sigma (_Qi \cdot [h_{t-1}, y_t] + b_i) \\ \tilde{S}_t= & \tanh (Q_S \cdot [h_{t-1}, y_t] + b_S) \end{aligned}$$

where $Q_i$ and $Q_S$ are weight matrices, and $b_i$ and $b_S$ are bias vectors. The cell state is then updated by combining the previous cell state and the new candidate cell state:

$$S_t = x_t *S_{t-1} + i_t *\tilde{S}_t$$

Finally, the output gate, a significant element, determines the output of the current cell state, using:

$$\begin{aligned} p_t= & \sigma (Q_o \cdot [h_{t-1}, y_t] + b_o) \\ h_t= & p_t *\tanh (S_t) \end{aligned}$$

where $Q_o$ is the weight matrix and $b_o$ is the bias vector. The following formulas explain how an LSTM network operates by utilizing the current input and the previous hidden state as input, meaning that LSTM networks can detect hidden dependencies in the data^58^.

#### Convolution neural network (CNN)

CNN applies convolution to extract features from the data. This type of neural network has been extensively employed in the last few years due to its characteristic feature learning power; it ranges from photo classification Through time prediction to defect detection^59^. The pooling and convolution layers are often included in combinations of two. At the convolution layer, many convolution cores perform a convolution operation on the input data while abstracting the feature of the inputted data. The pooling procedure eliminates the extra data characteristics; depending on the continuous convolution layer, the pooling layer is located between it. The pooling layer uses dimension reduction sampling and data compression to extract more valuable details. Equation (2) depicts the convolution process. The local feature of the convolution layer output is defined as:

2 $$\begin{aligned} w_i = f(x \otimes \omega _i + y_i) \end{aligned}$$

The activation function *f*(*a*) is defined as:

3 $$\begin{aligned} f(a) = \text {ReLU}(a) = {\left\{ \begin{array}{ll} 0, & \text {if } a \le 0 \\ a, & \text {if } a > 0 \end{array}\right. } \end{aligned}$$

Where a list of inputs of CNN is as follows: When *x* refers to the input of CNN; $w_i$ refers to the *i*-th local feature of the convolution layer output; $\otimes$ denotes the process of convolution. This paper sets this model’s activation function of $f(\cdot )$ as the nonlinear activation function ReLU and indicates ReLU as expression (3). *y* refers to the bias matrix, where$y_i$ stands for the bias in the first weight matrix and convolution kernel, while it indicates the convolution kernel, and *i* is the number of convolution kernels.

#### Recurrent neural network (RNN)

RNNs are built to record passed values because they are used in sequential or time series data. However, because of the vanished gradient problem, which hinders their memorization abilities over long sequences, their ability to capture long trends tends to be negatively affected. Recurrent neural systems are a specific type of neural network based on which it is possible to introduce hidden states and past outputs as inputs that exhibit dynamic temporal characteristics. In turn, the RNNs, based on feed-forward neural networks, embed the sequences of different lengths into their internal state (memory) and tied each neuron’s output to its input. An NN’s most basic structural concept is to copy connection weight configurations to zero to approximate specific neurons having no interconnections^60^^61^. For each timestep $t$, the activation $b^{\langle t \rangle }$ and the output $v^{\langle t \rangle }$ are expressed as follows:

1 $$\begin{aligned} b^{\langle t \rangle }= & g_1(Q_{bb} a^{\langle t-1 \rangle } + Q_{bz} z^{\langle t \rangle } + b_b) \end{aligned}$$

2 $$\begin{aligned} v^{\langle t \rangle }= & g_2( Q_{vb} a^{\langle t \rangle } + b_v) \end{aligned}$$

where $Q_{bz}$, $Q_{bb}$, $Q_{vb}$, $b_b$, and $b_v$ are coefficients that are shared temporally, and $g_1$ and $g_2$ are activation functions.

In the case of a recurrent neural network, the loss function $\mathscr {L}$ of all timesteps is defined based on the loss at every timestep as follows:

3 $$\begin{aligned} \mathscr {L}(\hat{v}, v) = \sum _{t=1}^{T_v} \mathscr {L}(\hat{v}^{\langle t \rangle }, v^{\langle t \rangle }) \end{aligned}$$

Back-propagation was carried out at each point in time. At timestep $T$, the derivative of the loss $\mathscr {L}$ with respect to the weight matrix $Q$ is expressed as follows:

4 $$\begin{aligned} \frac{\partial \mathscr {L}(T)}{\partial Q} = \sum _{t=1}^{T} \frac{\partial \mathscr {L}(T)}{\partial Q}(t) \end{aligned}$$

**Fig. 5:**
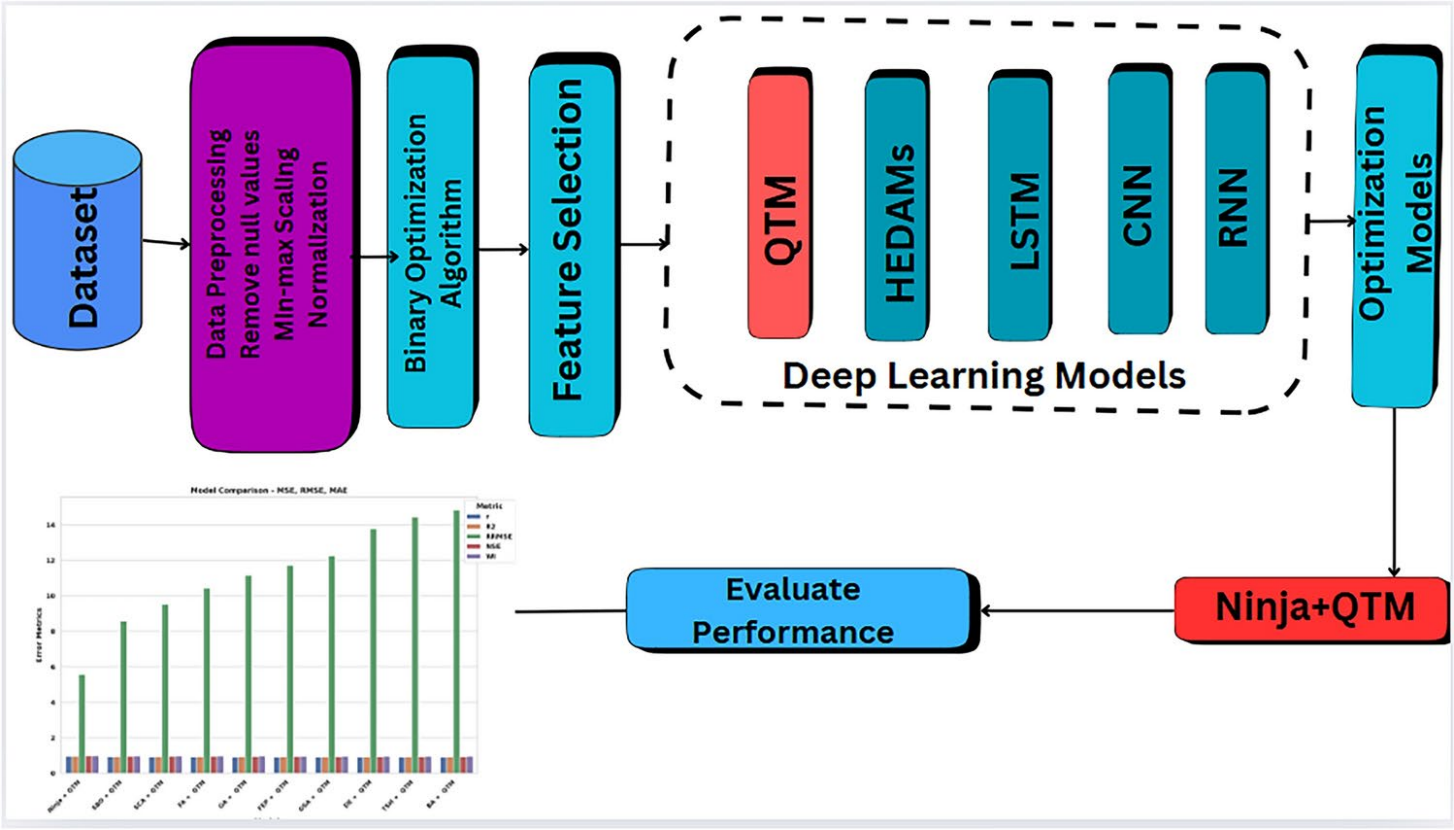
Block schematic of the proposed methodology.

## Proposed methodology

The proposed renewable energy forecasting methodology is shown in the flowchart depicted in Fig. 5. In this figure, the first phase is data preprocessing, in which the dataset is collected and preprocessed to remove outliers and handle missing values, and the data is cleaned. In addition, this phase includes data normalization that helps to eliminate the potential for bias toward outlying numbers. The second phase is feature selection, in which a novel feature selection algorithm is proposed based on the Ninja Optimization algorithms. The third phase is the optimization of the QTM prediction model. The optimization of this model is applied in terms of the proposed optimization algorithm. The fourth stage is forecasting renewable energy using the optimized model. The fifth stage is the evaluation and statistical analysis of the achieved prediction results.

### Quantum temporal model (QTM)

Quantum Temporal Models (QTMs) are a strategic development in predictive approaches that use quantum computing concepts to attain high accuracy in Renewable Energy Forecasting. The problems of time series datasets, especially short-term oscillations and long-term trends, like those shown in patterns of renewable energy across nations, are intended to be addressed by this state-of-the-art architecture. QTMs provide a revolutionary alternative to conventional models like Recurrent Neural Networks (RNNs), Long Short-Term Memory (LSTM), Convolutional Neural Networks (CNNs), and Hybrid Encoder-Decoder Attention Models (HEDAMs), which are limited in their ability to handle long-term dependencies and computational efficiency. QTMs can process several temporal relationships simultaneously by utilizing quantum superposition and entanglement, significantly improving computational speed and predictive accuracy. In contrast to LSTM, which necessitates substantial processing resources, and RNNs, which have trouble with vanishing gradients, QTMs effectively capture both short-term fluctuations and long-term trends with little training complexity. CNNs are good at extracting geographical information but can’t wholly comprehend time series data’s sequential dependencies. Similarly, HEDAMs still have scalability problems when working with large datasets, even when they employ attention techniques. QTMs are the best option for high-precision renewable energy prediction and other real-world applications needing speed, scalability, and accuracy because they use quantum-based computation to reshape the time series forecasting environment. As a computing platform, a quantum computer relies on principles of quantum mechanics for computation using a particle known as a qubit, an elementary unit for computation. They have a superb positional nature; they can occupy both 0 and 1 or any phase between 0 and 1, making representing a wide range of phases possible. Quantum circuit components are called quantum gates, essential for manipulating the qubits to perform a computing procedure. Hadarmard Gate, Controlled-NOT Gate, and Toffoli Gate all show the parallel computing nature of quantum computers. These properties are exploited by quantum algorithms so that their speed is dramatically superior to classical ones. For instance, Grover’s search algorithm provides a quadratic level speed to find a particular element out of N-elements in an unsorted list, a framework better than classical steps. Classical factoring algorithms are usually categorized to the highest degree, while quantum algorithms can offer widespread efficiency boosts for sub-exponential time factoring techniques. In the theory of quantum algorithms, the form of quantum states is vectors in a complex Hilbert space. At the same time, linear transformations define the changing of these states after applying quantum gates. Quantum circuits are generally based on quantum gates described by some form of rotations in the Hilbert space. For instance, the rotation gates for one-qubit can be defined in the following forms:

2 $$\begin{aligned} Y_a(2\theta ) = \begin{pmatrix} \cos (\theta ) & -i \sin (\theta ) \\ -i \sin (\theta ) & \cos (\theta ) \end{pmatrix} \end{aligned}$$

3 $$\begin{aligned} Y_b(2\theta ) = \begin{pmatrix} \cos (\theta ) & -\sin (\theta ) \\ \sin (\theta ) & \cos (\theta ) \end{pmatrix} \end{aligned}$$

4 $$\begin{aligned} Y_c(2\theta ) = \begin{pmatrix} e^{-i\theta } & 0 \\ 0 & e^{i\theta } \end{pmatrix} \end{aligned}$$

Because of these rotational components, the quantum circuits can provide a natural equivalent to the classical Fourier networks. Quantum computers have various implementation methods, including ion trap quantum computers, superconducting quantum computers, optical quantum computers, silicon photonics computers, and topological quantum computers. Ion trap quantum computers employ kept charged ions within electromagnetic fields as qubits, providing such advantages as excellent coherence interval, excellent gate fidelity, and possible extension. Superconducting quantum computers apply quantum principles like superposition and entanglement to provide solutions faster than traditional computers can. Topological quantum computers aim to achieve fault-tolerant quantum computation by encoding quantum information intrinsically resilient to decoherence and errors.

### Ninja optimization (NiOA) algorithm

The optimization approach presented in this study is referred to as the Ninja Optimization Algorithm (NiOA), is a meta-heuristic optimization technique that draws inspiration from ninja traits like agility and dexterity. To optimize the exploration and exploitation phases and facilitate both localized and global search in the best possible way, NiOA is methodically designed to address complex optimization issues. Each element of the created population represents an agent or solution in a given issue space that the Algorithm begins with. At each phase, the agents place themselves according to exploration, mutation and exploitation exploration tactics. These tactics, he said, provide a way to move away from the local optimum and towards a global optimum. In the process, the optimal answer is continuously adjusted, which improves team results, he continued. Below is the Algorithm of proposed optimization 1.

**Algorithm 1:**
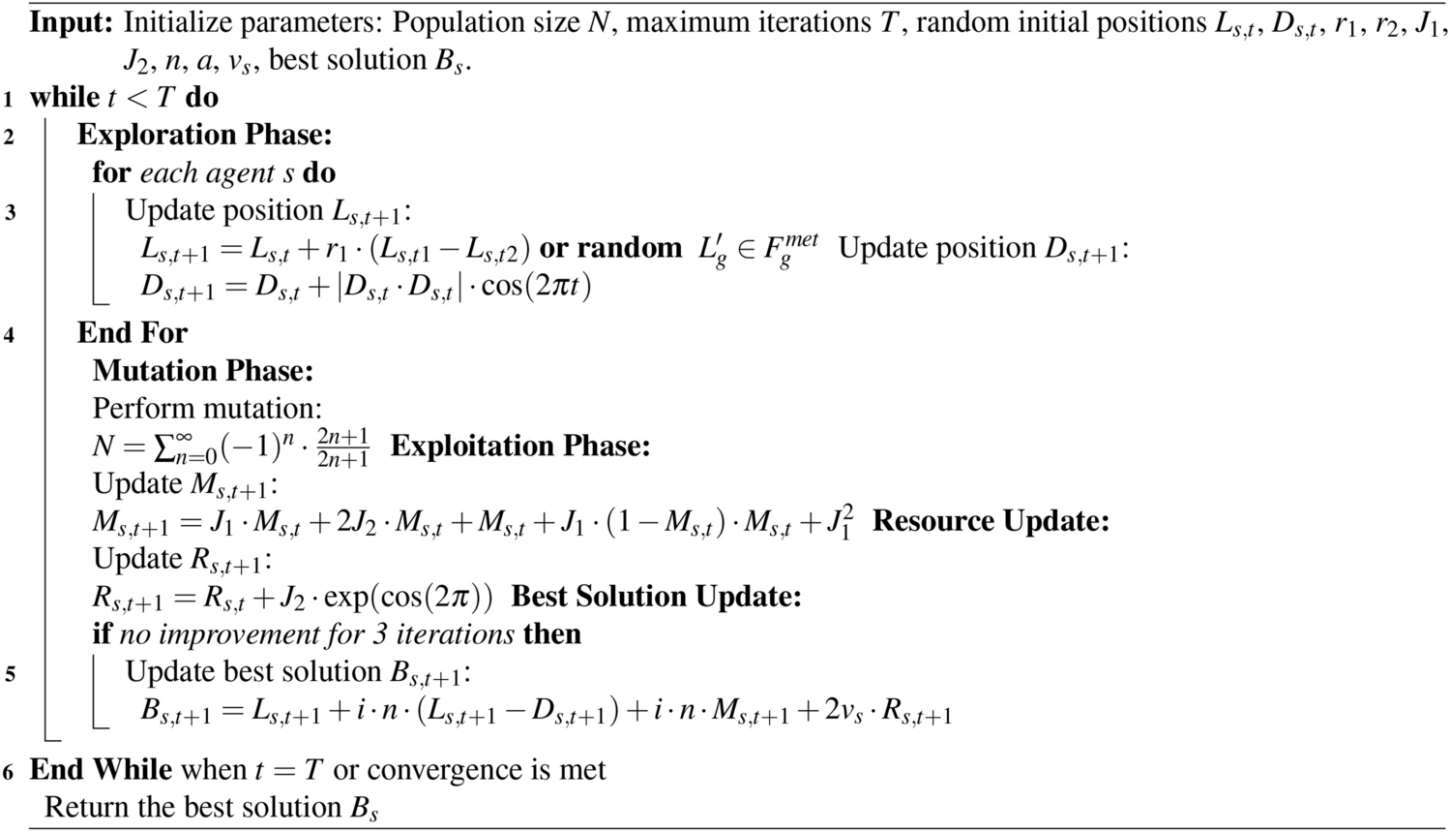
Ninja Optimization Algorithm (NiOA)

An improved method for feature selection of QTM parameters is the Ninja optimization algorithm. For feature selection, the Ninja makes use of the binary format. Problems with feature selection center on a narrow search area that only includes the binary numbers 0 and 1. Finding the relevance of a specific trait is the goal. In the binary Ninja technique proposed in this section, the continuing Ninja values are transformed to a binary [0, 1] format to conform to the feature selection procedure. Equations (5) and (6) describe the fundamental goal of this process, which is to use the Sigmoid function to transform the continuous data into binary data.

5 $$\begin{aligned} A_{k,T}^{*}= & {\left\{ \begin{array}{ll} 1 & \text {if } \sigma \left( A_{k,T}^{*}\right) \ge 0.5, \\ 0 & \text {otherwise.} \end{array}\right. } \end{aligned}$$

6 $$\begin{aligned} \text {Sigmoid}\left( A_{k,T}^{*}\right)= & \frac{1}{1 + e^{-10\left( A_{k}^{*k} - 0.5\right) }} \end{aligned}$$

Where $A_{k,T}^{*}$ represents the best solution at specific iteration $T$. This research explores how Ninja can enhance the tuning of QTM parameters. Optimizing QTM parameters is essential for maximizing performance and achieving the highest renewable energy prediction. Before using Ninja to adjust the values in QTM, it is crucial to determine the parameters that require optimization. The optimization of the deep learning models and the proposed QTM models is conducted using the provided and discussed optimization algorithm proposed in this section. The suggested optimization approach uses the principles of stealth, precision and activity from the film about ninja fighters to design an optimization procedure that solves high-dimensional optimization tasks while simultaneously being efficient. With efficient exploration and subsequent exploitation planning, NiOA guarantees it will be able to move in the problem space fluidly and purposefully, akin to the Japanese ninjas in their operations., which divide into two groups, as explained in the following sections.

### Exploration group

In the exploration phase, NiOA investigates diverse potential solutions across the search space to prevent getting trapped in local optima. The following position update equation governs the exploration behavior for a search agent $L_s(t + 1)$ at time step $t + 1$:

7 $$\begin{aligned} L_s(t + 1) = {\left\{ \begin{array}{ll} L_s(t) + r_1 \cdot (L_s(t_1) - L_s(t_2)), & \text {if specific conditions are met,} \\ \text {Random } L_s(t') \in FS(\text {met}), & \text {otherwise.} \end{array}\right. } \end{aligned}$$

Here, $r_1$ is a random factor that introduces variability in the movement of the search agent, while $t_1$ and $t_2$ refer to previous iterations of the Algorithm. This equation ensures that the Algorithm explores new regions of the search space, thereby avoiding stagnation. If certain predefined conditions are met (such as insufficient improvement in the fitness function), the Algorithm randomly selects values within the feasible solution space $FS(\text {met})$, further promoting exploration. In addition, the position of the second variable, $D_s(t + 1)$, is updated according to the following equation:

8 $$\begin{aligned} D_s(t + 1) = D_s(t) + |D_s(t) + r_2 \cdot D_s(t) |\cdot \cos (2\pi t), \end{aligned}$$

where $r_2$ is another random scaling parameter. The cosine function introduces oscillatory behavior, encouraging exploration by varying the magnitude and direction of the search agent’s movements. This periodic behavior helps NiOA avoid premature convergence by allowing for the exploration of both global and local areas of the search space.

### Mutation mechanism

NiOA includes a mutation mechanism to enhance the diversity of the explored solutions. The mutation operator introduces controlled randomness, which enables the Algorithm to escape from local optima and discover new potential solutions. The mutation equation is defined as:

9 $$\begin{aligned} N = \sum _{n=0}^{a} \frac{(-1)^n}{2n + 1} \cdot x \cdot (2n + 1), \end{aligned}$$

where $a$ is a randomly generated integer. This mutation mechanism applies a perturbation to the current solution, creating a more diverse set of candidate solutions for the next iteration. Using random integers and alternating signs ensures that the mutation effect is varied, enhancing the Algorithm’s ability to explore underexplored regions of the search space.

### Exploitation phase

Once promising regions of the search space have been identified, NiOA enters the exploitation phase, where the search intensifies around high-quality solutions. This phase aims to refine the solutions by focusing on a local search around the best candidates found during exploration. The update equation for the solution $M_s(t + 1)$ during exploitation is given by:

10 $$\begin{aligned} M_s(t + 1) = J_1M_s(t) + 2J_2 \cdot \big (M_s(t) + \big (M_s(t) + J_1\big )\big ) \cdot \Bigg (1 - \frac{M_s(t)}{M_s(t) + J_1}\Bigg )^2, \end{aligned}$$

where $J_1$ and $J_2$ are constants that control the intensification of the search. This equation encourages the Algorithm to focus on exploiting the local neighborhood around the best solutions. At the same time, the squared term modulates the step size, preventing over-exploitation and ensuring that the search remains adaptable.

### Solution update mechanism

The solution update in NiOA is designed to adapt dynamically as the search progresses. The solution $R_s(t+1)$ is updated using the following equation:

11 $$\begin{aligned} R_s(t + 1) = R_s(t) + (1 + R_s(t) + J_2) \cdot \exp (\cos (2\pi )), \end{aligned}$$

where $\exp (\cos (2\pi ))$ introduces non-linearity in the solution update, helping the Algorithm to adapt dynamically to changes in the fitness landscape. This update rule ensures that NiOA balances between intensifying the search around promising solutions and maintaining sufficient exploration to discover new, potentially better solutions.

### Stagnation handling and update

To prevent the Algorithm from stagnating when no improvement is observed over a certain number of iterations, NiOA applies the following update mechanism to generate new potential solutions:

12 $$\begin{aligned} B(s(t + 1)) = L_s(t + 1) + i \cdot n \cdot \big (L_s(t + 1) - D_s(t + 1)\big ) + i \cdot n \cdot \big (M_s(t + 1) + 2v_s \cdot R_s(t + 1)\big ), \end{aligned}$$

where $i$, $n$, and $v_s$ are parameters that control the intensity and direction of the update. This equation ensures that if the Algorithm is stuck in a local optimum, a more aggressive update is applied to the solution, increasing the chances of escaping the local optimum and continuing the search for a global solution.

### Hyperparameters tuning

The parameters used in NiOA are critical to its performance and must be carefully tuned. The following ranges are typically used for these parameters:

- $a \in [6, 10]$—controls the mutation behavior.
- $v_1 \in [0, 1]$—a scaling factor for exploitation.
- $r_2 \in [0, 1]$—a random factor for exploration.
- $r_3 \in [0, 2]$—a random factor for exploitation.
- $J_1 \in [0, 2]$—adjusts the intensification step size.
- $J_2 \in [0, 2]$—controls the dynamic adaptation of the solution.
- $n \in [0, 2]$—controls the update during stagnation.

These parameters offer flexibility to NiOA because they enable participation in a wide range of optimization problems. A tendency of parameters can greatly influence the result; thus, parameters are normally tuned according to the problem solved. Table1 explains hyperparameters tuning.

**Table 1:** Hyperparameters Tuning.

Parameter	Value range
1. Quantum circuit parameters	
Number of qubits (n)	6
Depth of quantum circuit	2-layers
2. Optimization parameters	
Learning rate ($\alpha$)	0.0001
Optimizer	Ninja
Batch size	16

### Fitness function

The fitness function is used to gauge how well the optimizer solutions perform. The number of features chosen and the classification error rate affect the fitness function. A solution is deemed suitable if it chooses a subset of characteristics with fewer selected features and a lower classification error rate. The quality of each solution will be assessed using the following formula.

13 $$\begin{aligned} Z_{n} = p_{1} V(D)+ p_{2} \dfrac{|l|}{| z|} \end{aligned}$$

Where $l$ is the number of selected features, $z$ is the total number of features, $p_1 \in [0,1]$, $p_2 = 1 - p_1$ controls the significance of the number of selected features for a population of size $n$. The classification error rate, and $V(D)$ is the classifier’s error rate.

### Fine-tuning

Fine-tuning^62^ is one of the most popular transfer learning techniques in neural network operations. Switching knowledge from the generative to the discriminative model provided a fantastic generalization and was first applied in^63^. The first pipeline consisted of a pre-trained network, and a randomly initialized layer was used for the final layer and the classifier layer. The concept of fine-tuning a plan is similar to the present concept. While it is not common to replace the last layer classifier with a randomly initialized one, as I mentioned before, we do this quite often when some of the layers of a pre-trained neural network can be fine-tuned. However^64^, the question of when deciding which layers of a network model to fine-tune is raised now. Usually, a deep neural network’s last couple of layers are trained, but the preceding layers are initialized with preprocessing values and are known as frozen. This propels this approach: joining size-constrained data sets with proof that the lower layers of a deep neural network are more general in properties. The higher-level layers provide more specific, task-specific properties attached to objects, whereas such properties generally extend to a broader range of tasks. As noted in^57^, there are numerous examples of fine-tuning approaches that have achieved significant success while masking the fine-tuning of the last few layers; the process of selecting layers that are still fine-tuning remains largely the manual process that often requires a great deal of trial and error. Moreover, some recent studies dispute the idea that the features of the early or middle layer should be shared. Based on these empirical observations and the absence of rules and guidelines for choosing fine-tunable layers, we decided to address the problem from the perspective of its representation as an optimization problem. We tried to solve it using a widely known optimization meta-heuristic algorithm.

## Experimental results

The section highlights a novel feature selection method that helps select appropriate features from the dataset to improve prediction accuracy. A statistical analysis supports the innovative approach which leads to the optimized DL model results presentation.

### Feature selection results

**Fig. 6:**
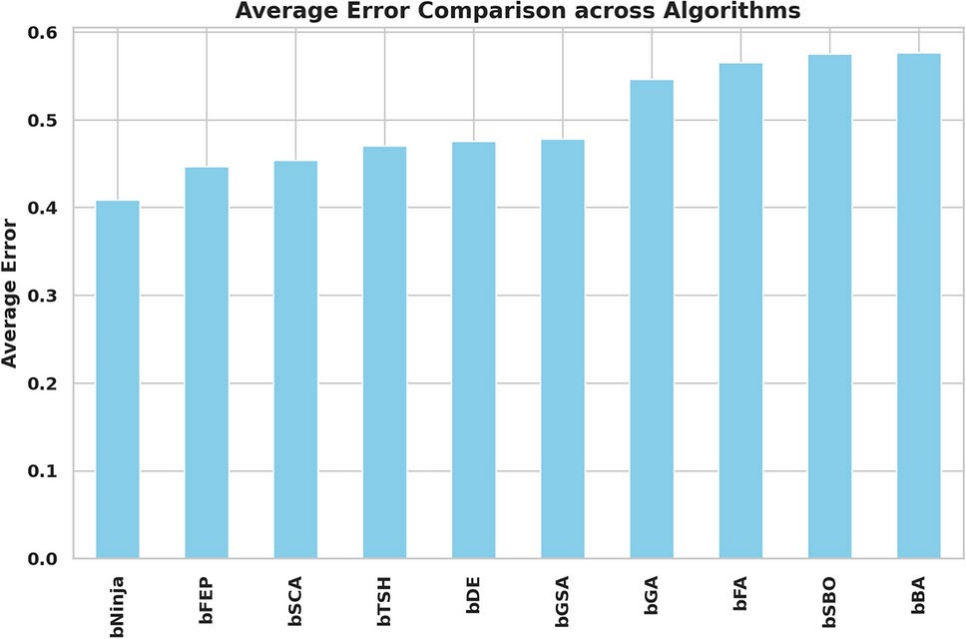
The average error of the feature selection results based on the proposed and other feature selection methods.

**Fig. 7:**
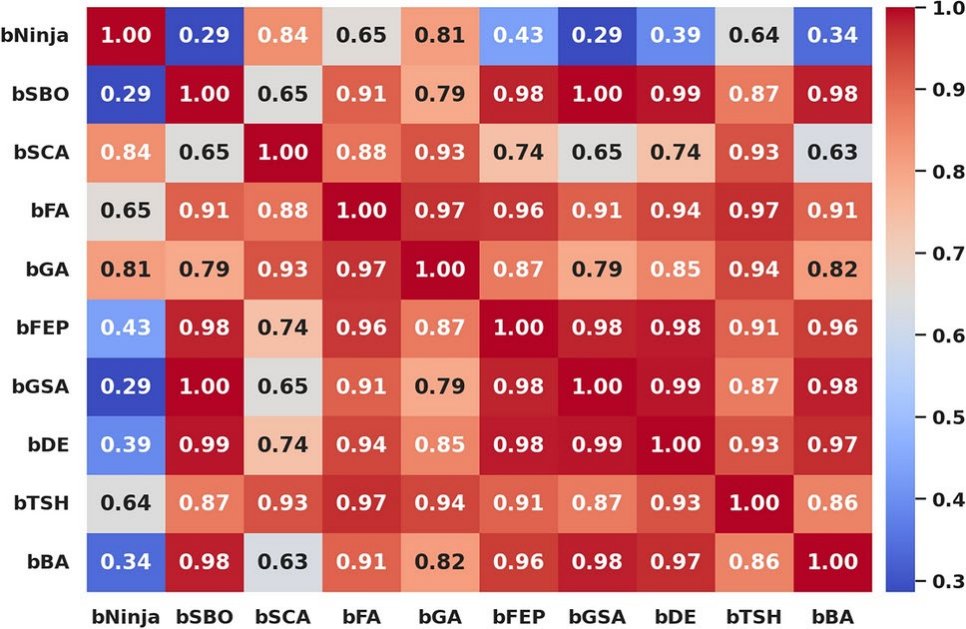
Feature heatmap matrix.

**Fig. 8:**
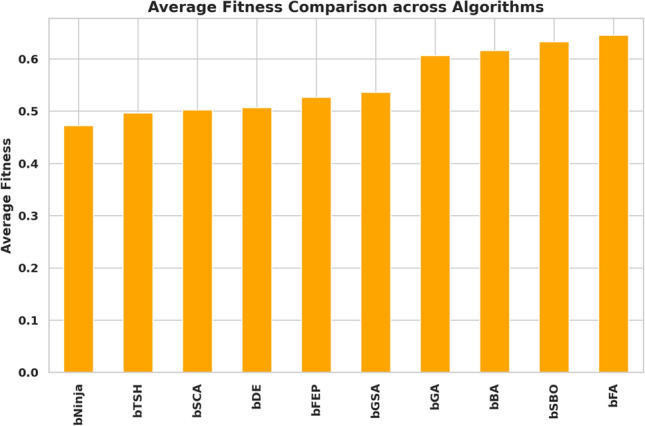
Average Fitness comparison across algorithms.

**Fig. 9:**
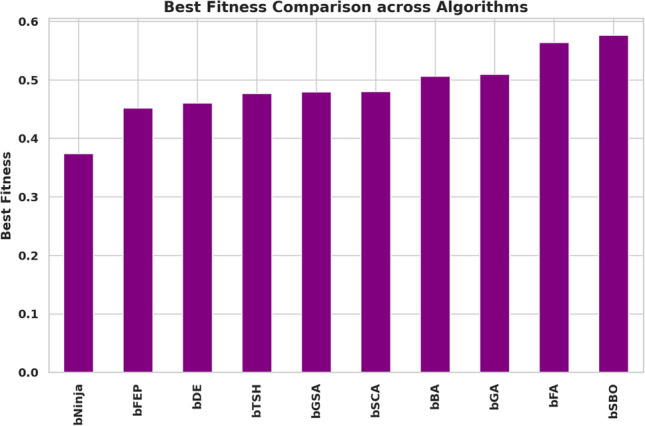
Best fitness comparison across algorithms.

**Fig. 10:**
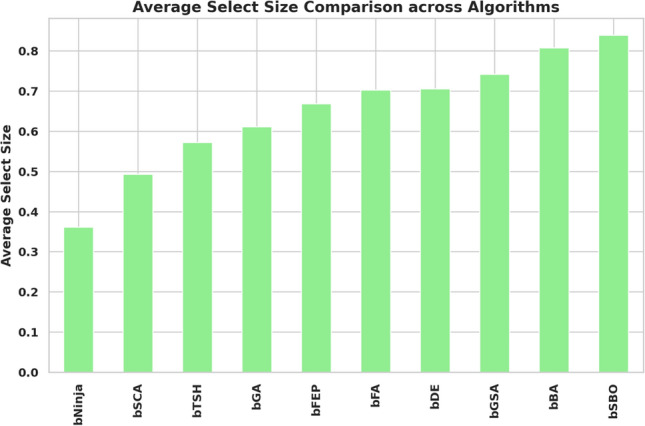
Average select size comparison across algorithms.

**Fig. 11:**
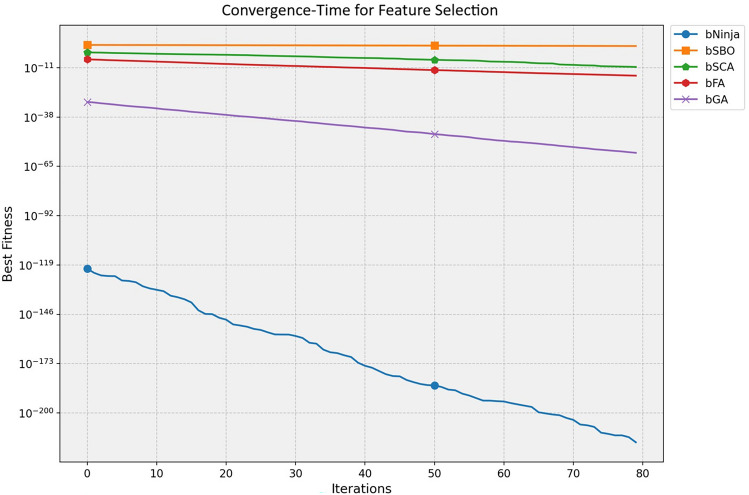
Convergence-time for feature selection.

The study performed in-depth research using ten binary versions of optimization algorithms named bNinja, bSBO, bSCA, bFA, bGA, bFEP, bGSA, bDE, bTSH and bBA. The feature selection results are assessed from the metrics shown in Table 2, which matter considerably. This set of metrics includes best fitness, worst fitness, average error, average fitness size, standard deviation, and average fitness. Experiments were performed using the configuration parameters presented in the previous section. Table 3 presents the evaluation of the proposed feature selection method compared to current feature selection methods in the literature. The test results using fitness, select size, and error measurements show without doubt that the proposed feature selection approach works effectively. This solution achieved a superior fitness score of 0.374, surpassing the algorithm goals and providing excellent accuracy while not imposing any size limitation on the selection at an average of 0.361. The average error of 0.409 indicates that the number of errors caused by the model has decreased according to the chosen features. Yet, given that the mistake rate is not zero, there is space for enhancement. The average fitness score of 0.472 for the chosen attributes is relatively high. An std fitness of 0.294 shows a wide range of values for the specified features’ fitness metrics. This indicates that the features may vary greatly depending on the characteristics. Finally, the worst fitness value of 0.472 is encouraging because it indicates that the feature selection technique has not chosen any features with a meager fitness score. The proposed feature selection method has selected a subset of characteristics with a high fitness score and low error rate. However, the forecasted fitness value and the error rate have a lot of percentage differences, so the impact shows that there is still a chance to improve training. A reasonable assessment of these parameters is possible using a comparatively simple and plausible approach, which can be the basis for developing additional research and improving this approach. Thirdly, Fig. 6 demonstrates the relative average errors in feature selection using the proposed and competitor methods. From the Figure above, the average error of the proposed feature selection algorithm is the lowest; the algorithm outperforms the various algorithms in identifying the right characteristics pertinent to the prediction activity. The scatter figure proves that the feature selection algorithm proposed in the paper is the most accurate in multi-dataset analysis, confirming its efficiency and applicability. The possibility of the algorithm in terms of selection of features: the algorithm helps to determine which of the features are more critical for the prediction task, and reduction of features, which enables us to reduce the number of input parameters without loss of accuracy, also proves the reliability of the algorithm, establishing a proper basis for application of the algorithm in the field of the predictive model. Furthermore, it can be seen from the graphic qualitatively how the proposed feature selection algorithm differs from other algorithms used in the study, where it stands, and what the differences are. This knowledge can be used to determine the best feature selection algorithms for a given prediction problem, considering the dataset and prediction method in question. The proposed approach achieves the lowest average error. It surpasses the other algorithms in selecting relevant features for the prediction job, as shown by a plot of the average error of the algorithms employed in feature selection compared to the proposed algorithm. This indicates that the proposed approach is not only efficient but also a trustworthy, helpful tool for feature selection, which can be used to improve the high precision and productivity of the prediction algorithms of a large group of applications and does not leave any doubt of effectiveness. The heatmap of features in the dataset is shown in Fig. 7. Renewable energy time series prediction correlates many features in a dataset, and performing a heatmap analysis is an effective way to study these correlations. Heatmap analysis can also generate more reliable and accurate predictive models and identify some relationships and potential confounding factors. Figure 8 compares average fitness values across different algorithms. This is depicted in the bar chart below, where the orange bars represent the average fitness of the slopes created by these algorithms. From the results obtained, algorithms such as the BFA and bSBO yield the most significant average fitness, meaning that these algorithms balance the exploration and exploitation stages well. On the other hand, there is a lower score obtained by algorithms such as bNinja and bTSH, which may cause some drawbacks in their inherent optimization capabilities. Based on this analysis, Fig. 9 compares the best fitness values observed from different algorithms. The purple bar chart shows the highest accuracy of the algorithms in this experiment. As seen in the first Figure, both bFA and bSBO algorithms can provide the best fitness value and, therefore, indicate better solutions than other algorithms. On the other hand, the bNinja algorithm has quite a low performance in this case, which gives a clear perception of the disparities in the performance of the above algorithms. Furthermore, in Fig. 10, we provide an analogous view only for the average selection size over all algorithms. The green bar chart below illustrates how well each algorithm identifies the selection size. A greater average selection size is also observed for bSBO and bBA, which strengthens past results, indicating the superior ability of these approaches for resource exploitation. However, the bNinja algorithm has an average here, which is relatively lower than other algorithms and supports the inferiority regarding both fitness measures. All these results together give a generalized picture of the strengths and weaknesses of the individual algorithm in terms of different performance factors. The performance of different feature selection algorithms in fitness function improvement throughout iterations is represented in Fig. 11. Through its operation, bNinja demonstrates powerful optimization ability, resulting in sustained reductions of fitness function values. In contrast, other algorithms such as bSBO, bSCA, and bFA maintain almost constant fitness values, suggesting their limited ability to enhance performance or their tendency to get trapped in local minima. The bGA algorithm shows slight improvement but is much slower than bNinja, reflecting its limited efficiency in finding optimal solutions. This chart indicates that Ninja is the most effective algorithm for feature selection, demonstrating superior optimization capability compared to other methods. These results show that this experiment’s best algorithm for handling feature selection is bNiOA.

**Table 2:** Formulas of the metrics used in evaluating the feature selection results.

Metric	Formula
Best fitness	$\min _{i=1}^{M} S_i^*$
Worst fitness	$\max _{i=1}^{M} S_i^*$
Average error	$\frac{1}{M} \sum _{i=1}^{M} \frac{1}{N} \sum _{i=1}^{N} \text {mse}(\overline{V_i} - V_i)$
Average fitness	$\frac{1}{M} \sum _{i=1}^{M} S_i^*$
Average fitness size	$\frac{1}{M} \sum _{i=1}^{M} \text {size}(S_i^*)$
Standard deviation	$\sqrt{\frac{1}{M-1} \sum _{i=1}^{M} (S_i^* - \text {Mean})^2}$

**Table 3:** Evaluation metrics used in assessing the proposed feature selection method.

Metric	bNinja	bSBO	bSCA	bFA	bGA	bFEP	bGSA	bDE	bTSH	bBA
Average error	0.40908	0.57498	0.45428	0.56508	0.54648	0.44678	0.47838	0.47558	0.47008	0.57628
Average select size	0.36188	0.83878	0.49258	0.70298	0.61088	0.66838	0.74218	0.70518	0.57188	0.80788
Average fitness	0.47228	0.63318	0.50248	0.64538	0.60648	0.52658	0.53658	0.50678	0.49688	0.61638
Best fitness	0.37408	0.57628	0.48028	0.56408	0.50978	0.45178	0.47968	0.46028	0.47718	0.50608
Worst fitness	0.47258	0.65598	0.55648	0.66168	0.62488	0.56978	0.55938	0.57028	0.54488	0.60768
Standard deviation fitness	0.29458	0.46618	0.30778	0.44208	0.40748	0.35938	0.36958	0.32748	0.30868	0.41518

### Prediction results

The experiment was performed to stress the significance of improving the feature selection process on the prediction results. The current data went through categorization employing deep learning models based on the attributes outlined by the bNinja framework, a widely recognized and practical framework in deep learning. This technique significantly improved the feature space and the models’ overall fitting. The prediction results for various deep learning algorithms and post-feature selection are summarized in Table 4. Table 5 contains information about several deep learning structures such as QTM, HEDAM, LSTM, RNN, and CNN. The table also shows the Mean Squared Error (MSE), Root Mean Squared Error (RMSE), Mean Absolute Error (MAE) and correlation coefficient (r). QTM gives the lowest MSE of 0.016 and the lowest RMSE of 0.02, outcompeting all other models. The correlation coefficient, or r value for QTM, is 0.76, which, on any scale, can be classified as an excellent means of prediction. Besides, the current study has shown that QTM has better computational efficiency, where the model has been fitted within a shorter time compared to other models, which are the most commonly used. This paper presents NiOA optimization algorithms that enhance QTM prediction model performance to generate additional accurate results. NiOA optimization methods aim to enhance the QTM model by adjusting its parameters to produce improved data alignment, which leads to better predictions. Table 6 presents the performance metrics of different models after applying the proposed optimization Ninja. The best performance of the models is presented by the Ninja + QTM model pair when conducting a comparative analysis according to different criteria. It has the least MSE, RMSE, MAE and RRMSE.

**Table 4:** Statistical performance metrics and their equations.

Metric	Equation
Mean square error	$\text {MSE} = \dfrac{1}{n} \sum _{j=1}^{n} \left( F_j - Z_j \right) ^2$
Root mean square error (RMSE)	$\displaystyle \text {RMSE} = \sqrt{\frac{1}{s} \sum _{m=1}^{s} (x_m - \hat{x}_m)^2}$
Mean absolute error (MAE)	$\displaystyle \text {MAE} = \frac{1}{s} \sum _{m=1}^{s} \left| x_m - \hat{x}_m \right|$
Mean bias error (MBE)	$\displaystyle \text {MBE} = \frac{1}{s} \sum _{m=1}^{s} (\hat{x}_m - x_m)$
Correlation coefficient (r)	$\displaystyle r = \frac{\sum _{m=1}^{s} (x_m - \overline{x})(\hat{x}_m - \overline{\hat{x}})}{\sqrt{\sum _{m=1}^{s} (x_m - \overline{x})^2} \sqrt{\sum _{m=1}^{s} (\hat{x}_m - \overline{\hat{x}})^2}}$
Coefficient of determination (R^2^)	$\displaystyle R^2 = 1 - \frac{\sum _{m=1}^{s} (x_m - \hat{x}_m)^2}{\sum _{m=1}^{s} (x_m - \overline{x})^2}$
Relative root mean square error (RRMSE)	$\displaystyle \text {RRMSE} = \frac{\sqrt{\frac{1}{s} \sum _{m=1}^{s} (x_m - \hat{x}_m)^2}}{\overline{x}}$
Nash-sutcliffe efficiency (NSE)	$\displaystyle \text {NSE} = 1 - \frac{\sum _{m=1}^{s} (x_m - \hat{x}_m)^2}{\sum _{m=1}^{s} (x_m - \overline{x})^2}$
Willmott index of agreement (WI)	$\displaystyle \text {WI} = 1 - \frac{\sum _{m=1}^{s} (x_m - \hat{x}_m)^2}{\sum _{m=1}^{s} \left( \left| \hat{x}_m - \overline{x} \right| + \left| x_m - \overline{x} \right| \right) ^2}$

**Table 5:** Performance metrics for different models.

Models	MSE	RMSE	MAE	MBE	r	R^2^	RRMSE	NSE	WI
QTM	0.0161	0.0219	0.1940	0.0984	0.7625	0.7751	23.01	0.8501	0.8855
HEDAM	0.0903	0.1868	0.2370	0.1594	0.7226	0.7352	25.21	0.8378	0.8362
LSTM	0.1046	0.3115	0.2758	0.1791	0.6755	0.6881	26.05	0.7803	0.7434
RNN	0.1189	0.3554	0.3146	0.1988	0.5327	0.5453	26.63	0.7561	0.6439
CNN	0.1346	0.3695	0.3286	0.3391	0.4941	0.5067	27.06	0.7302	0.5775

**Table 6:** Model performance metrics after proposed optimization.

Models	MSE	RMSE	MAE	MBE	r	R^2^	RRMSE	NSE	WI
Ninja + QTM	3.09E−06	3.07E−05	2.92E−05	0.000107228	0.946080	0.951610	5.565489	0.976254	0.979377
SBO + QTM	0.000114385	0.000140887	0.000597287	0.000934072	0.918957	0.928813	8.574589	0.953245	0.967520
SCA + QTM	0.000131815	0.000147113	0.000600811	0.000934683	0.917390	0.922921	9.521145	0.949563	0.960125
FA + QTM	0.000157308	0.000147834	0.000603866	0.000935416	0.915768	0.921299	10.432197	0.947604	0.957520
GA + QTM	0.000167918	0.000171034	0.000614203	0.000946033	0.903600	0.920442	11.152798	0.942041	0.955549
FEP + QTM	0.000178053	0.000186048	0.000622817	0.000949451	0.902703	0.918256	11.709071	0.938805	0.952310
GSA + QTM	0.000182024	0.000199084	0.000630648	0.000957262	0.901885	0.914113	12.256628	0.928479	0.953943
DE + QTM	0.000252189	0.000213632	0.000638480	0.000964952	0.899570	0.909970	13.772998	0.925874	0.948733
TSH + QTM	0.000289146	0.000218024	0.000641299	0.000972763	0.898302	0.908855	14.433742	0.923269	0.947298
BA + QTM	0.000395989	0.000332753	0.000647251	0.000984602	0.894660	0.907641	14.833742	0.921223	0.946360

**Fig. 12:**
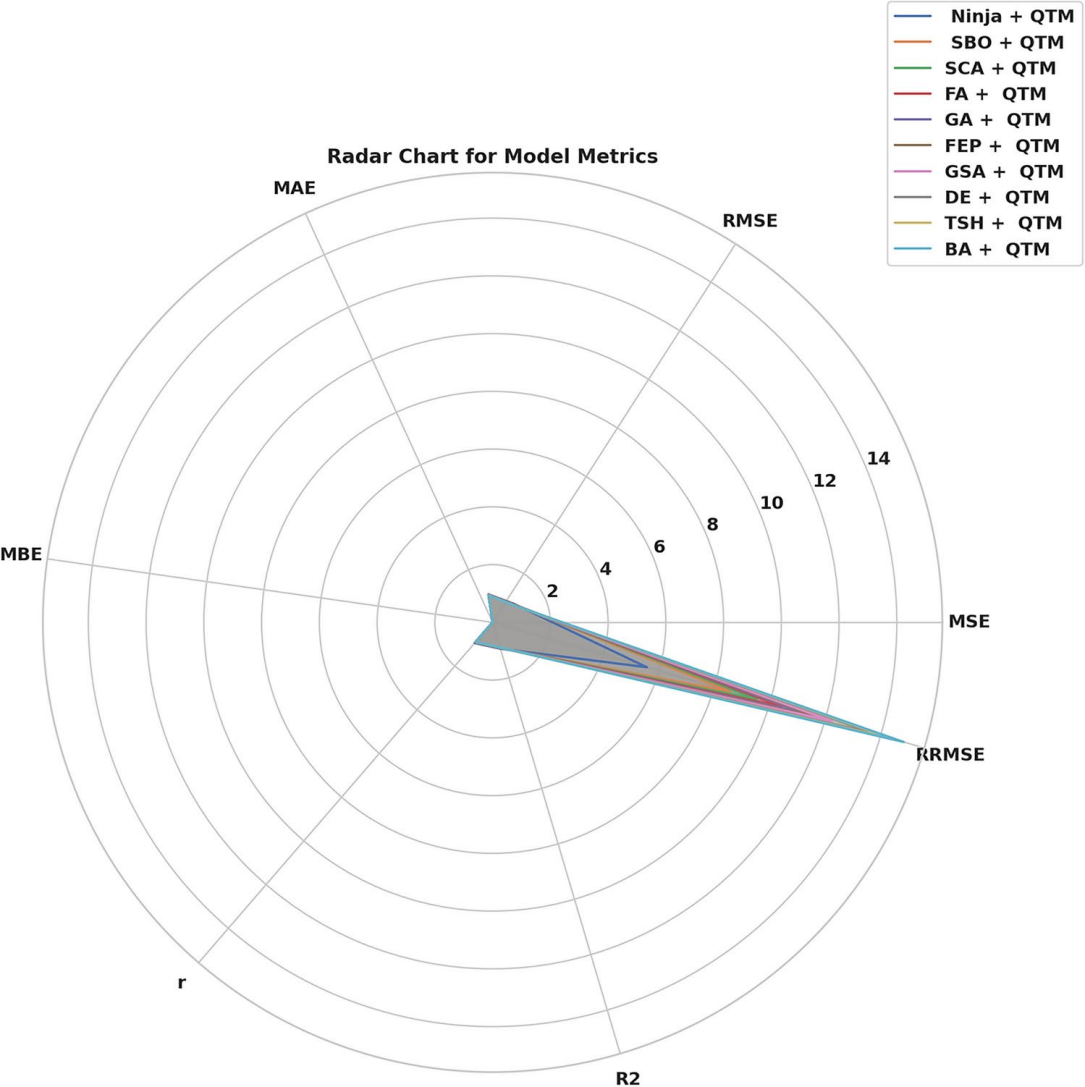
Radar plot of model performance metrics.

**Fig. 13:**
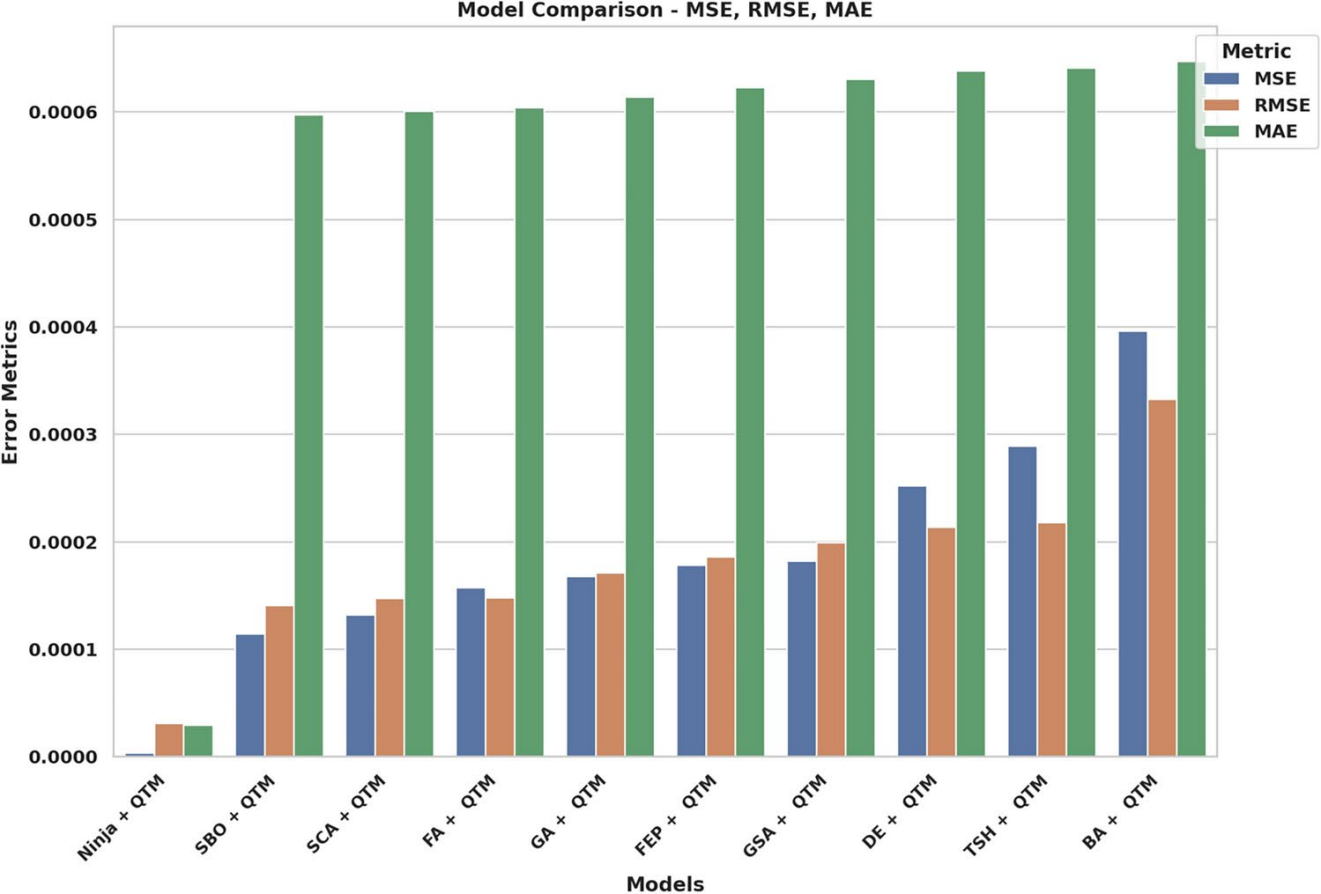
Bar plot of model performance metrics depend on MSE, RMSE, MAE.

**Fig. 14:**
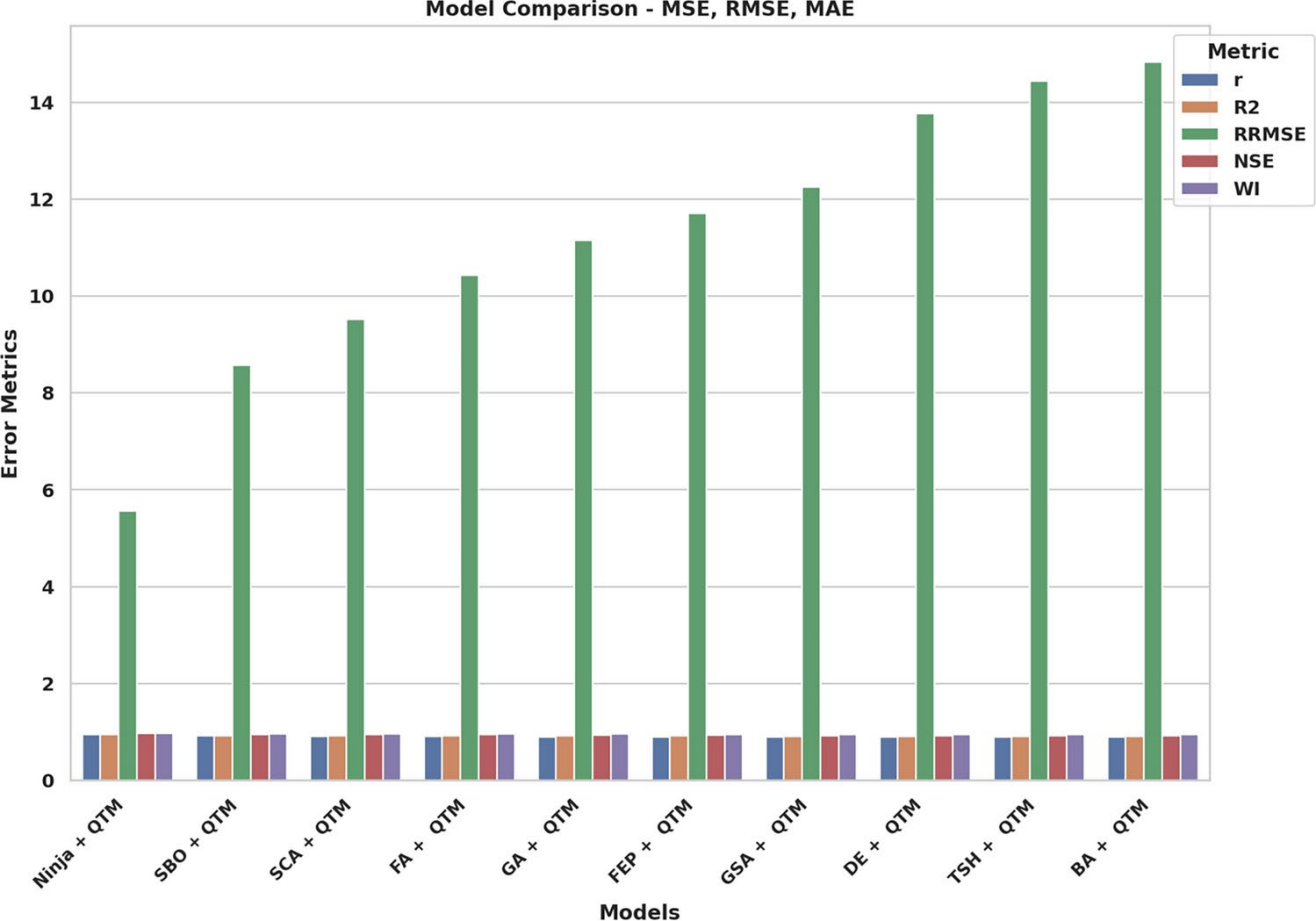
Bar plot of model performance metrics depend on error metrics.

It gives the maximum value of correlation coefficient (r = 0.946), coefficient of determination (R^2^ = 0.9516) and weighted index of agreement (WI = 0.9794 which shows that the regularity between the anticipated and primary value is rather good. The results indicate that BA + QTM and TSH + QTM models demonstrate less effective performance since they produce increased errors and weaker efficiency scores than the other models. The results demonstrate that the Ninja + QTM model achieves superior accuracy and reliability compared to other measured models, thus making it a strong option for renewable energy forecasting tasks. The RMSE analysis of QTM models through Different Optimization Tactics can be found in Fig. 12. NiOA demonstrates superiority over earlier approaches when evaluating RMSE values. The visual presentation shows NiOA achieves higher precision in adjusting model parameters than other optimization methods thus delivering better accuracy in weather prediction. A comparison of error metrics from several hybrid models is shown in Fig. 13, demonstrating the predictive accuracy of each model in anticipating renewable energy. The performance of several optimization strategies combined with QTM, such as Ninja-QTM, SBO-QTM, FA-QTM, and others, is depicted in the bar chart. Lower MSE, RMSE, and MAE values indicate prediction accuracy and dependability. Higher error metrics indicate more variability in prediction accuracy, whereas models with lower error values-like Ninja-QTM-show better stability. The best model for projecting renewable energy is chosen with the help of this study, which guarantees better resource management and decision-making. Figure 14 provides a comparative analysis of multiple hybrid models utilizing different optimization techniques combined with QTM, focusing on key performance metrics: r, R^2^, RRMSE, NSE, and WI. The measurement tools evaluate the predictive models’ accuracy, reliability factors, and consistency in renewable energy predictions.

**Fig. 15:**
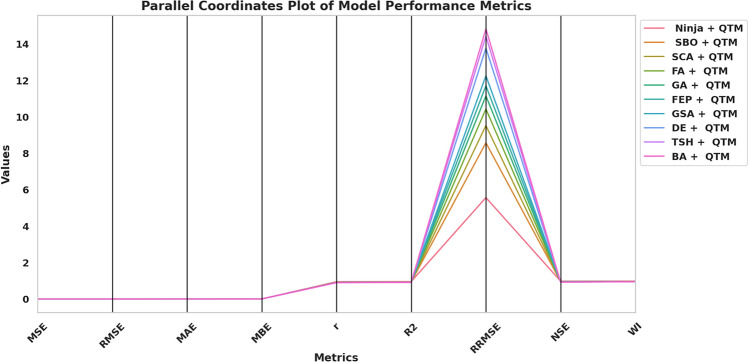
Parallel plot of performance comparison of models across evaluation metrics.

**Fig. 16:**
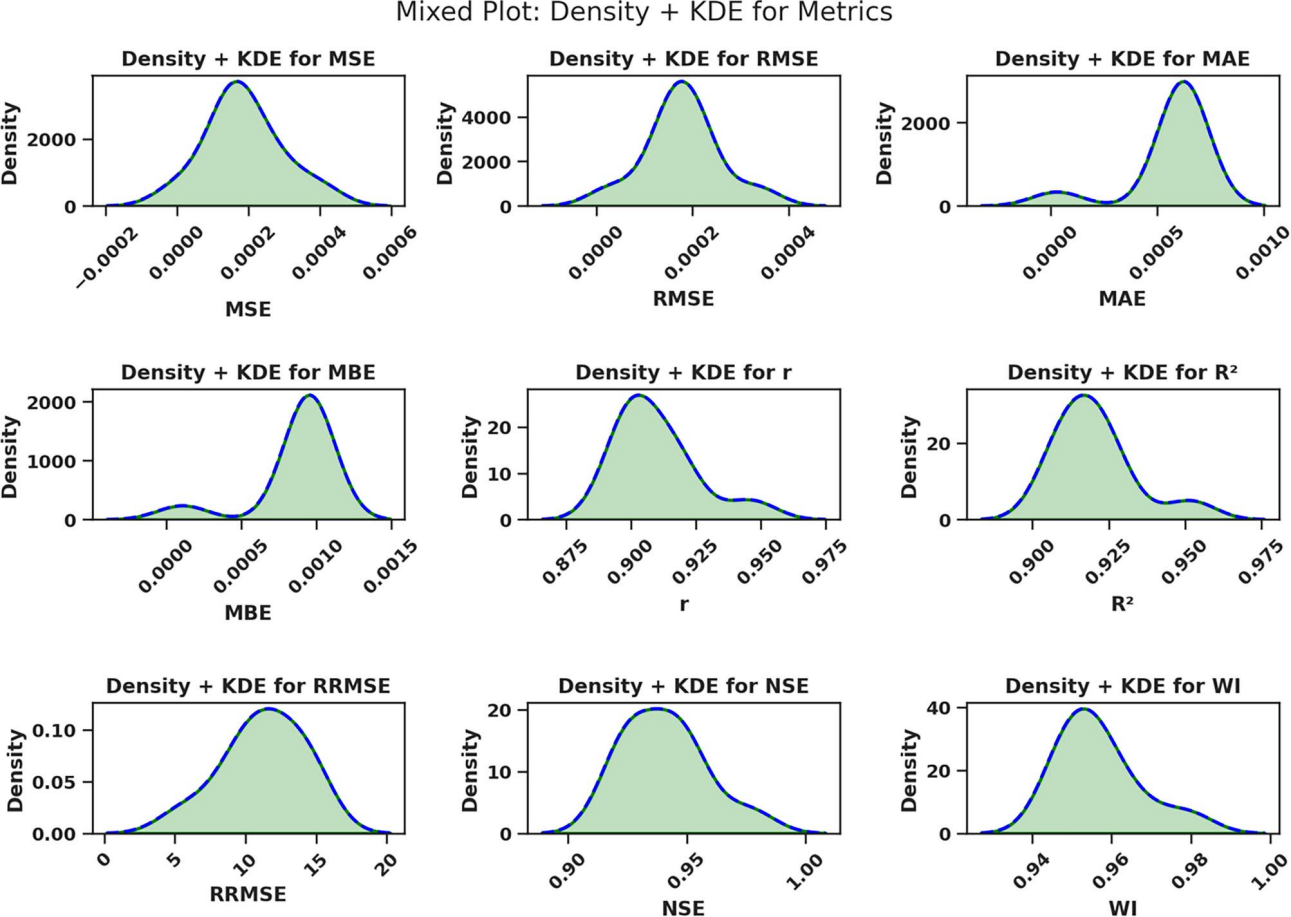
KDE plot for various performance metrics for the optimized models.

**Fig. 17:**
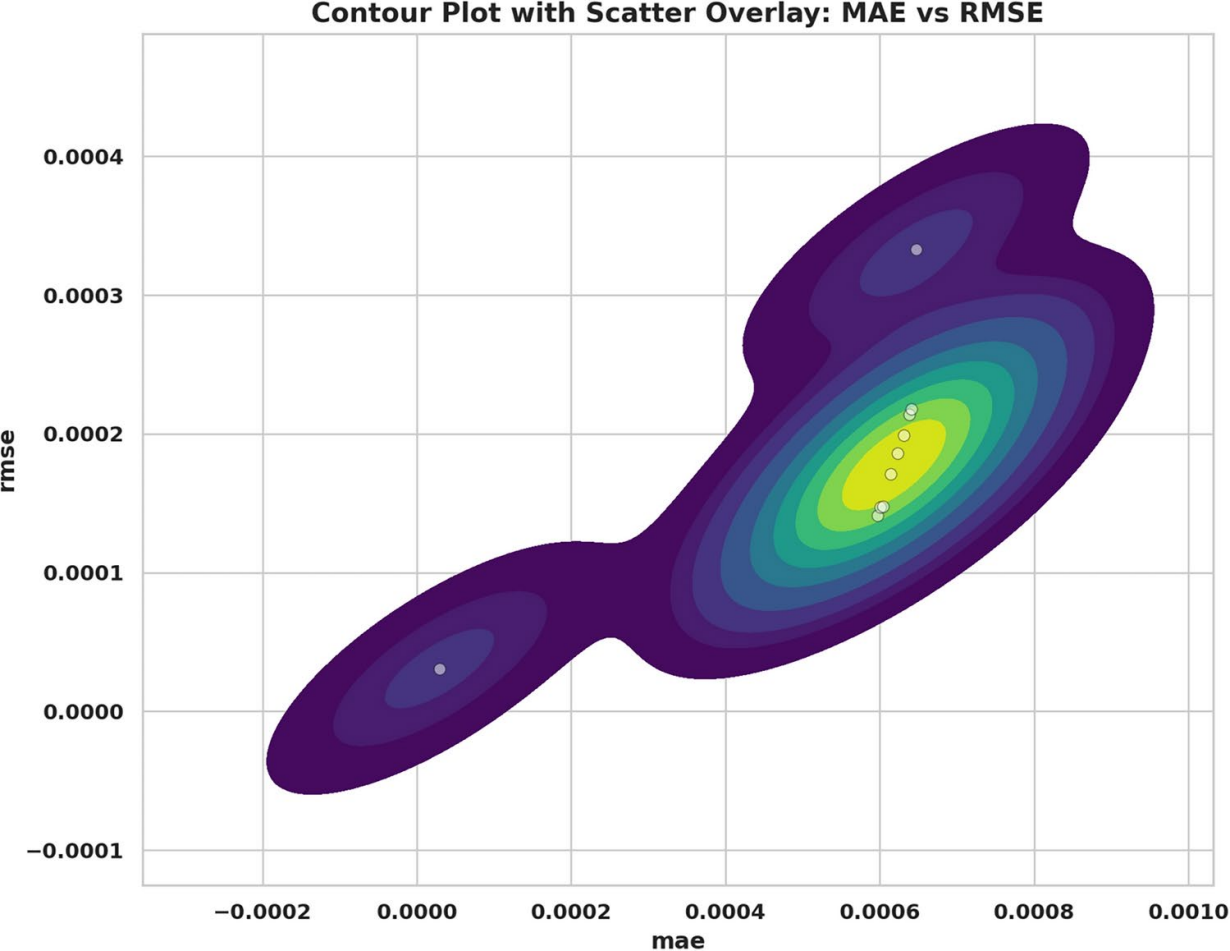
Contour and scatter plot: the correlation of MAE and RMSE.

**Fig. 18:**
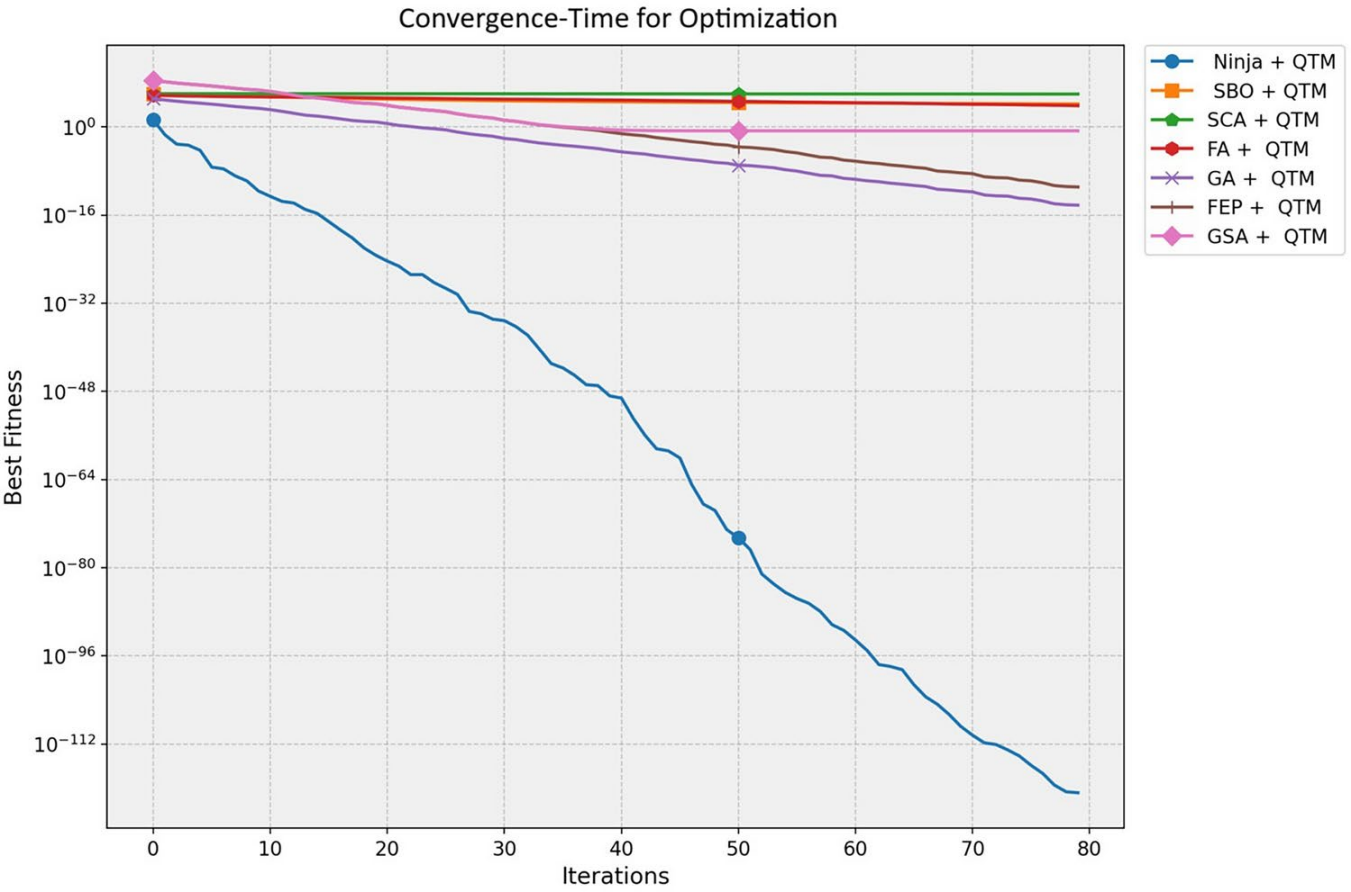
Convergence-time for optimization.

The RRMSE values in the chart display significant variations throughout models as they represent predictive accuracy through lower numbers. The Ninja + QTM model presents the best RRMSE results, indicating high performance and minimal error transmission. Conversely, models such as BA + QTM and TSH + QTM demonstrate significantly higher RRMSE values, indicating reduced efficiency and more significant deviations in predictions. Other statistical measures, including correlation coefficient (r), coefficient of determination (R^2^), Nash-Sutcliffe efficiency (NSE), and Willmott’s Index (WI), remain relatively stable across models, suggesting that while error magnitudes differ, overall predictive reliability is maintained. Model predictive accuracy in Ninja + QTM and SBO + QTM is substantial based on their high R^2^ and NSE values, but the predictive capability of BA + QTM remains weak. The evaluation process confirms the need to pick optimization techniques that maintain prediction precision and system stability. Models with lower RRMSE and higher R^2^ and NSE values, such as Ninja + QTM, are more effective for enhancing forecasting accuracy in renewable energy applications, ensuring optimal resource allocation and improved decision-making in sustainable energy management. Figure 15 illustrates how the Ninja + QTM model outperforms the other models in a parallel plot. One of the plot’s peculiarities is its capacity to perform a thorough comparative analysis of multiple models simultaneously, considering multiple performance criteria. Each line denotes a distinct model type, and the plots on the axes show how well each model performed on each metric, allowing us to compare models and identify their strengths and weaknesses quickly. Figure 16 presents Kernel Density Estimates (KDE) for the assessment performance indicators of the improved models. Visual presentation of error behavior and accuracy predictions becomes possible through these charts because they display an entire statistical distribution of each statistic. The density of observed values is depicted by the KDE curves, which also show the overall distribution of the data and the most common metric values. Metrics like MSE, RMSE, and MAE show unimodal distributions with peaks concentrated around tiny error values, indicating that the models typically produce low prediction errors. In the meantime, performance metrics such as R2, NSE, and WI show density curves that are concentrated close to higher values, indicating both robust predictive performance and a good correlation between expected and actual values. The optimization approach’s stability and consistency levels can be examined by examining KDE curve shapes and widths because high consistency corresponds to sharp peaks, and wide spreads indicate unpredictable behavior. The possibility of outliers or situations where the model’s performance deviates under particular circumstances is indicated by long tails or modest asymmetries in particular distributions. Figure 17 combines a contour plot and a scatter plot to provide a comprehensive view of the results achieved for the MAE and RMSE tests. The curves plot the density of this relation, and the grey scales represent the intensity or fluctuation of the values. Small dots within the plot add further explanation, showing where individual points of interest within the range are placed. This visualization is a powerful tool for understanding and interpreting model performance. This can be seen from the plot where the spot with the denser area is located close to the origin, coinciding with the MAE and RMSE values being closest to zero. This indicates that the model or data tied to the plot is excellent for the region where it is implemented with little or no error. Such analysis can be of considerable help in assessing the results obtained from the models and definition of the directions, which may need additional enhancement. By comparing the given RMSE value graphs, we can conclude that the RMSE value range of our suggested method is the smallest among the approaches. This means that on the average of the results of every analyzed dataset, the suggested approach is superior to other approaches. The charts also make it easy to see how similar and different approaches are. This fact is also confirmed by the histogram of the distribution of the RMSE, which shows a consistently high performance of the suggested method. Most of the RMSE values by the proposed approach are clustered around a small band of values, confirming that the recommended method maintains superior accuracies throughout the investigated datasets. Since some datasets yielded very high error rates, the RMSE for other methods is comparatively more dispersed. From this, the posited approach is more specific regarding datasets and more stable and reliable than the options under consideration. Histograms and plots of the RMSE values show that the recommended methodology outperforms these approaches. As this histogram shows, the recommended approach produces consistently high accuracy with a dispersion of RMSE values within a minimal range of values, highlighting its efficiency. These findings can guide the selection of DL models as they guide how to build more accurate and efficient models. Figure 18 demonstrates that the Ninja + QTM algorithm achieves superior performance compared to its alternatives based on optimization task results. Ninja + QTM effectively finds optimal solutions because its fitness function value drops exponentially in optimization. The performance metrics of SBO + QTM, SCA + QTM, and FA + QTM remain steady across the trial runs because these algorithms demonstrate inadequate exploration capabilities for fitness function minimization. The minimization improvements shown by GA + QTM and FEP + QTM proceed slowly when compared to the increased speed of Ninja + QTM. The combination of Ninja + QTM strikes the best equilibrium between effective search capabilities and optimal solution attainment, creating a strong selection for optimizing problems where fast fitness function minimization is needed.

### Discussions

Electrical energy demand expands fast because of worldwide industrialization together with urbanization processes. Numerous studies have shown fossil fuels cannot sustain electricity production because they result in dangerous environmental consequences and global warming effects. Renewable energy sources (RES) are the most sustainable, climate-friendly solution, prompting international attention. Analysts predict renewable energy will take control of electricity generation, which will exceed half of the total capacity by 2050, according to research in^65^.Many scientific investigations work to enhance the implementation and optimization of solar power and wind power, representing the principal renewable energy sources currently in use. Researchers have examined different facets of renewable energy by assessing power predictions identifying proper equipment dimensions, and studying power distribution processes. Research widely uses artificial intelligence techniques above physical-based models because AI provides more accurate predictions. This research studies AI-based predictions for solar and wind power variations according to the information presented in Table 7. Studies have established that artificial intelligence models primarily serve short-term energy forecasting needs, yet engineers face ongoing problems with accuracy improvement and error reduction.Feature selection and optimization technologies at an advanced level improve the research’s accuracy of solar and wind energy forecasting. Experimental data confirms that NiOA provides the best optimization solution, decreasing prediction errors and strengthening reliability. Time-series prediction tasks benefit from the NiOA implementation within QTM networks, creating a balanced relation between short-term and long-term forecasting data. The proposed models’ effectiveness has been validated through Radar and Parallel plots that both validate the statistical reliability of the produced results. The proposed framework presents a dedicated forecasting system for renewable energy required by environmental approaches seeking climate change reduction and sustainable energy development.

**Table 7:** Optimization techniques and models for renewable energy forecasting.

Author(s)	Model	Advantages	Disadvantages
Zhou et al.^33^	Dual-stage attention LSTM + VMD	Outperforms traditional models (XGBoost, CNN-LSTM, Transformer)	Requires further tuning of metaheuristic algorithms
López et al.^34^	LSTM + correlation matrix	Performs better than ARMA, NARX, NIO (R, MSE, MAPE)	Suffers from premature convergence
Suo et al.^35^	BiGRU + IChOA	Enhanced parameter tuning and performance	Prone to premature convergence
Wei et al.^36^	DBN + SSO	Improved wind prediction capability	High computational cost
Zhu et al.^37^	MOECO	Outperforms traditional multi-objective optimization methods	Scalability challenges for large datasets
Wu et al.^38^	JADE + TFT	Ensures stable and reliable wind speed fusion	Increased computational complexity
Chen et al.^39^	DAE-GRU + MOSMA	High accuracy and minimal bias	Requires extensive parameter tuning
Wang et al.^40^	CSSOA + LSTM	Enhanced wind forecasting using chaos sequences	Requires Gaussian mutation tuning
Ewees et al.^41^	HBO + LSTM	Outperforms GA in optimization	Prone to premature convergence
Hossain et al.^42^	CNN-GRU + Fully connected NN	High accuracy for short-term wind power predictions	Computational overhead
Bou et al.^43^	BiLSTM	Captures SE fluctuations with high accuracy	Performance varies under different conditions
Al et al.^44^	CNN-LSTM-transformer	Maintains computational efficiency for large datasets	Requires extensive data preprocessing
Djaafari et al.^45^	BDSCA-LSTM	Effective for DNI estimation in arid regions	Climate-specific applicability
Teferra et al.^46^	Fuzzy-PSO	Improved forecast accuracy with error correction	Computationally intensive
Gupta et al.^47^	EEMD-GA-LSTM	Addresses nonlinearity in solar data	Needs parameter fine-tuning
Wu et al.^49^	CNN-LSTM (spatiotemporal)	Outperforms standalone CNN/LSTM models	Requires high-quality input data
Neshat et al.^50^	Quaternion CNN + BiLSTM + AVMD	Improved accuracy in short-term and long-term forecasts	Complex architecture and training time
Ai et al.^44^	SSA + VMD + Sample entropy + LSTM	Robust performance in short-term wind speed forecasting	Requires extensive preprocessing
Diaa Salman et al.^51^	CNN-LSTM-transformer	Enhanced accuracy for solar power forecasting	Requires optimal selection of optimizers
Bashir et al.^52^	CNN-ABiLSTM, CNN-transformer-MLP	Improved accuracy for short and long-term solar/wind forecasting	High computational cost
Zhang et al.^53^	BiTCN-BiGRU + Multi-head attention	Better wind power forecasting using PCC and CEEMDAN	Increased model complexity
Liu et al.^54^	TCN-LSTM + Tensor concatenate	Reduced computational cost with high accuracy	Requires preprocessing with SG filtering
Proposed model	QTM + NiOA	Higher convergence speed and a better balance between exploration and exploitation in the search process to avoid local optima	

## Conclusion

Predictive power estimation of renewable energy generation stabilizes power grid operations and optimizes administrative procedures. Independently deploying machine learning methods for renewable energy fails to generate beneficial results because of the unpredictable nature of renewable power outputs. A new binary bNinja algorithm is the proposed solution when selecting essential features for achieving more precise predictions. The proposed solution is evaluated regarding its performance alongside other feature selection strategies. The research offers an upgraded renewable energy forecasting approach based on QTM with NiOA. Sound preprocessing methodologies filter data inputs to supply essential information for model implementation to the procedure that models the data. The proposed algorithm achieves high prediction accuracy with R^2^ at 95.15% when utilized on Kaggle renewable energy forecasting data to forecast daily energy production while delivering a minimum RMSE of 0.00003. The research results using multiple performance indicators demonstrated that the proposed method outperforms current leading optimization methods while showing strong resistance against system variations. The proposed algorithms should tackle additional datasets to support their general applicability across Constrained engineering and classification and feature selection domains.

## Data Availability

The data that support the findings of this study are openly available at [https://www.kaggle.com/datasets/henriupton/wind-solar-electricity-production].

## References

[1] Bali Swain, R., Karimu, A. & Gråd, E. Sustainable development, renewable energy transformation and employment impact in the eu. *Int. J. Sustain. Dev. World Ecol.* **29**, 695–708. 10.1080/13504509.2022.2078902 (2022).

[2] IEA-International Energy Agency. Renewable energy market update-may 2022. https://www.iea.org/reports/renewable-energy-market-update-may-2022 (2022). Accessed 5 June 2022.

[3] Bedewy, B. A. H. & Al-Timimy, S. R. A. Estimate suitable location of solar power plants distribution by gis spatial analysis. *Civ. Eng. J.* **9**, 1217–1229 (2023).

[4] Alrikabi, N. Renewable energy types. *J. Clean Energy Technol.* **2**, 61–64. 10.7763/jocet.2014.v2.92 (2014).

[5] Alanazi, M. A., Aloraini, M., Islam, M., Alyahya, S. & Khan, S. Wind energy assessment using weibull distribution with different numerical estimation methods: A case study. *Emerg. Sci. J.* **7**, 2260–2278 (2023).

[6] Couto, A. & Estanqueiro, A. Wind power plants hybridised with solar power: A generation forecast perspective. *J. Clean. Prod.* **423**, 138793. 10.1016/j.jclepro.2023.138793 (2023).

[7] Ahilan, T., Yoganand, S. & Prasad, D. An improved metaheuristic method-based neural network for predicting wind turbine power. *Cybern. Syst.* **54**, 1424–1445 (2023).

[8] Guchhait, R. & Sarkar, B. Increasing growth of renewable energy: A state of art. *Energies* **16**, 2665. 10.3390/en16062665 (2023).

[9] De Silva, T. et al. Hydropower operation in future power grid with various renewable power integration. *Renew. Energy Focus* **43**, 329–339 (2022).

[10] Kumar, R. et al. Prospects of renewable energy scenario in india. In *Renewable Energy: Accelerating the Energy Transition*, 15–31 (Springer, 2023).

[11] Ntuli, M., Dioha, M., Ewim, D. & Eloka-Eboka, A. Review of energy modelling, energy efficiency models improvement and carbon dioxide emissions mitigation options for the cement industry in south africa. *Mater. Today Proc.* **65**, 2260–2268. 10.1016/j.matpr.2022.07.093 (2022).

[12] Mwanzia, D. et al.*A study of solar variability and its effects on Earth’s Climate*. Ph.D. thesis, University of Nairobi (2021).

[13] Jalili, M., Sedighizadeh, M. & Fini, A. S. Optimal operation of the coastal energy hub considering seawater desalination and compressed air energy storage system. *Therm. Sci. Eng. Progress* **25**, 101020. 10.1016/j.tsep.2021.101020 (2021).

[14] Khadidja, B., Dris, K., Boubeker, A. & Noureddine, S. Optimisation of a solar tracker system for photovoltaic power plants in saharian region, example of ouargla. *Energy Procedia* **50**, 610–618. 10.1016/j.egypro.2014.06.075 (2014).

[15] Saxena, N. et al. Deep learning approach for wind power forecasting. In *Advances in Communication, Devices and Networking: Proceedings of ICCDN 2021*, 355–367 (Springer, 2022).

[16] Gaamouche, R., Chinnici, M., Lahby, M., Abakarim, Y. & Hasnaoui, A. E. Machine learning techniques for renewable energy forecasting: A comprehensive review. *Comput. Intell. Tech. Green Smart Cities* **1**, 3–39. 10.1007/978-3-030-96429-0 (2022).

[17] Bull, S. R. Renewable energy today and tomorrow. *Proc. IEEE* **89**, 1216–1226. 10.1109/5.940290 (2001).

[18] Lai, J.-P., Chang, Y.-M., Chen, C.-H. & Pai, P.-F. A survey of machine learning models in renewable energy predictions. *Appl. Sci.* **10**, 5975. 10.3390/app10175975 (2020).

[19] Sharma, A. K. et al. Role of metaheuristic approaches for implementation of integrated mppt-pv systems: a comprehensive study. *Mathematics* **11**, 269. 10.3390/math11020269 (2023).

[20] Gusain, C., Tripathi, M. M. & Nangia, U. Study of meta-heuristic optimization methodologies for design of hybrid renewable energy systems. *Therm. Sci. Eng. Progress* **39**, 101711. 10.1016/j.tsep.2023.101711 (2023).

[21] Shuaibu Hassan, A., Sun, Y. & Wang, Z. Optimization techniques applied for optimal planning and integration of renewable energy sources based on distributed generation: Recent trends. *Cogent Eng.* **7**, 1766394. 10.1080/23311916.2020.1766394 (2020).

[22] Liu, G., Wang, C., Qin, H., Fu, J. & Shen, Q. A novel hybrid machine learning model for wind speed probabilistic forecasting. *Energies* **15**, 6942 (2022).

[23] Zhang, J. et al. Deterministic and probabilistic prediction of wind power based on a hybrid intelligent model. *Energies* **16**, 4237 (2023).

[24] Meenal, R. et al. Weather forecasting for renewable energy system: A review. *Arch. Comput. Methods Eng.* **29**, 2875–2891. 10.1007/s11831-021-09695-3 (2022).

[25] Solano, E. S., Dehghanian, P. & Affonso, C. M. Solar radiation forecasting using machine learning and ensemble feature selection. *Energies* **15**, 7049. 10.3390/en15197049 (2022).

[26] Olatomiwa, L. et al. A support vector machine-firefly algorithm-based model for global solar radiation prediction. *Sol. Energy* **115**, 632–644. 10.1016/j.solener.2015.03.015 (2015).

[27] Lu, J. et al. Investigation of landslide susceptibility decision mechanisms in different ensemble-based machine learning models with various types of factor data. *Sustainability* **15**, 13563 (2023).

[28] Zheng, X., Qi, X., Liu, H., Liu, X. & Li, Y. Deep neural network for short-term offshore wind power forecasting. In *2018 OCEANS-MTS/IEEE Kobe Techno-Oceans (OTO)*, 1–5 (IEEE, 2018).

[29] Huang, C.-M., Chen, S.-J., Yang, S.-P. & Chen, H.-J. One-day-ahead hourly wind power forecasting using optimized ensemble prediction methods. *Energies* **16**, 2688 (2023).

[30] Elbedwehy, S., Hassan, E., Saber, A. & Elmonier, R. Integrating neural networks with advanced optimization techniques for accurate kidney disease diagnosis. *Sci. Rep.* **14**, 21740. 10.1038/s41598-024-71410-6 (2024). 39289394 10.1038/s41598-024-71410-6 PMC11408592

[31] Rafati, A., Joorabian, M., Mashhour, E. & Shaker, H. R. High dimensional very short-term solar power forecasting based on a data-driven heuristic method. *Energy* **219**, 119647 (2021).

[32] Yin, R., Li, D., Wang, Y. & Chen, W. Forecasting method of monthly wind power generation based on climate model and long short-term memory neural network. *Glob. Energy Interconnect.* **3**, 571–576 (2020).

[33] Zhou, X. et al. Wind power forecast based on variational mode decomposition and long short term memory attention network. *Energy Rep.* **8**, 922–931 (2022).

[34] López, G. & Arboleya, P. Short-term wind speed forecasting over complex terrain using linear regression models and multivariable lstm and narx networks in the andes mountains, ecuador. *Renew. Energy* **183**, 351–368 (2022).

[35] Suo, L. et al. Wind speed prediction by a swarm intelligence based deep learning model via signal decomposition and parameter optimization using improved chimp optimization algorithm. *Energy* **276**, 127526. 10.1016/j.energy.2023.127526 (2023).

[36] Wei, Y. et al. Deep belief network with swarm spider optimization method for renewable energy power forecasting. *Processes* **11**, 1001 (2023).

[37] Zhu, Q., Jiang, F. & Li, C. Time-varying interval prediction and decision-making for short-term wind power using convolutional gated recurrent unit and multi-objective elephant clan optimization. *Energy* **271**, 127006. 10.1016/j.energy.2023.127006 (2023).

[38] Wu, B. & Wang, L. Two-stage decomposition and temporal fusion transformers for interpretable wind speed forecasting. *Energy* **288**, 129728. 10.1016/j.energy.2023.129728 (2024).

[39] Chen, W., Zhou, H., Cheng, L. & Xia, M. Prediction of regional wind power generation using a multi-objective optimized deep learning model with temporal pattern attention. *Energy* **278**, 127942. 10.1016/j.energy.2023.127942 (2023).

[40] Wang, J., Zhu, H., Zhang, Y., Cheng, F. & Zhou, C. A novel prediction model for wind power based on improved long short-term memory neural network. *Energy* **265**, 126283. 10.1016/j.energy.2022.126283 (2023).

[41] Ewees, A. A., Al-qaness, M. A., Abualigah, L. & Abd Elaziz, M. Hbo-lstm: Optimized long short term memory with heap-based optimizer for wind power forecasting. *Energy Convers. Manage.* **268**, 116022. 10.1016/j.enconman.2022.116022 (2022).

[42] Hossain, M. A., Chakrabortty, R. K., Elsawah, S. & Ryan, M. J. Very short-term forecasting of wind power generation using hybrid deep learning model. *J. Clean. Prod.* **296**, 126564 (2021).

[43] Bou-Rabee, M. A., Naz, M. Y., Albalaa, I. E. & Sulaiman, S. A. Bilstm network-based approach for solar irradiance forecasting in continental climate zones. *Energies* **15**, 2226. 10.3390/en15062226 (2022).

[44] Al-Ali, E. M. et al. Solar energy production forecasting based on a hybrid cnn-lstm-transformer model. *Mathematics* **11**, 676. 10.3390/math11030676 (2023).

[45] Djaafari, A. et al. Hourly predictions of direct normal irradiation using an innovative hybrid lstm model for concentrating solar power projects in hyper-arid regions. *Energy Rep.* **8**, 15548–15562. 10.1016/j.egyr.2022.10.402 (2022).

[46] Teferra, D. M., Ngoo, L. M. & Nyakoe, G. N. Fuzzy-based prediction of solar pv and wind power generation for microgrid modeling using particle swarm optimization. *Heliyon* **9**, 1–10 (2023). 10.1016/j.heliyon.2023.e12802 PMC9871071 36704286

[47] Gupta, A. & Gupta, K. Short term solar irradiation prediction framework based on eemd-ga-lstm method. *Strategic Plann. Energy Environ.* **1**, 255–280. 10.13052/spee1048-5236.4132 (2022).

[48] Aljanad, A., Tan, N. M., Agelidis, V. G. & Shareef, H. Neural network approach for global solar irradiance prediction at extremely short-time-intervals using particle swarm optimization algorithm. *Energies* **14**, 1213. 10.3390/en14041213 (2021).

[49] Wu, Q., Guan, F., Lv, C. & Huang, Y. Ultra-short-term multi-step wind power forecasting based on cnn-lstm. *IET Renew. Power Gener.* **15**, 1019–1029 (2021).

[50] Neshat, M., Nezhad, M. M., Mirjalili, S., Piras, G. & Garcia, D. A. Quaternion convolutional long short-term memory neural model with an adaptive decomposition method for wind speed forecasting: North aegean islands case studies. *Energy Convers. Manage.* **259**, 115590 (2022).

[51] Salman, D., Direkoglu, C., Kusaf, M. & Fahrioglu, M. Hybrid deep learning models for time series forecasting of solar power. *Neural Comput. Appl.* **36**, 9095–9112. 10.1007/s00521-024-09558-5 (2024).

[52] Bashir, T., Wang, H., Tahir, M. & Zhang, Y. Wind and solar power forecasting based on hybrid cnn-abilstm, cnn-transformer-mlp models. *Renew. Energy* **239**, 122055. 10.1016/j.renene.2024.122055 (2025).

[53] Zhang, X. et al. Short-term wind power prediction based on iceemdan decomposition and bitcn-bigru-multi-head self-attention model. *Electr. Eng.* **1**, 1–18. 10.1007/s00202-024-02638-8 (2024).

[54] Liu, S., Xu, T., Du, X., Zhang, Y. & Wu, J. A hybrid deep learning model based on parallel architecture tcn-lstm with savitzky-golay filter for wind power prediction. *Energy Convers. Manage.* **302**, 118122. 10.1016/j.enconman.2024.118122 (2024).

[55] Barjasteh, A., Ghafouri, S. H. & Hashemi, M. A hybrid model based on discrete wavelet transform (dwt) and bidirectional recurrent neural networks for wind speed prediction. *Eng. Appl. Artif. Intell.* **127**, 107340. 10.1016/j.engappai.2023.107340 (2024).

[56] El-Kenawy, E.-S.M. et al. Nioa: A novel metaheuristic algorithm modeled on the stealth and precision of Japanese ninjas. *J. Artif. Intell. Eng. Pract.* **1**, 17–35. 10.21608/jaiep.2024.386693 (2024).

[57] Cong, F., Hu, W., Huo, Q. & Guo, L. A comparative study of attention-based encoder-decoder approaches to natural scene text recognition. In *2019 International Conference on Document Analysis and Recognition (ICDAR)*, 916–921. 10.1109/ICDAR.2019.00151 (IEEE, 2019).

[58] Singla, P., Duhan, M. & Saroha, S. Solar irradiation forecasting by long-short term memory using different training algorithms. In *Renewable Energy Optimization, Planning and Control: Proceedings of ICRTE 2021, Volume 1*, 81–89. 10.1007/978-981-16-4663-8_7 (Springer, 2022).

[59] Zang, H. et al. Hybrid method for short-term photovoltaic power forecasting based on deep convolutional neural network. *IET Gen. Transm. Distrib.* **12**, 4557–4567. 10.1049/iet-gtd.2018.5847 (2018).

[60] Asghar, R., Fulginei, F. R., Quercio, M. & Mahrouch, A. Artificial neural networks for photovoltaic power forecasting: A review of five promising models. *IEEE Access*10.1109/ACCESS.2024.3420693 (2024).

[61] Yazdan, M. M. S., Saki, S. & Kumar, R. Untangling energy consumption dynamics with renewable energy using recurrent neural network. *Analytics* **2**, 132–145. 10.3390/analytics2010008 (2023).

[62] Hassan, E., Saber, A. & Elbedwehy, S. Knowledge distillation model for acute lymphoblastic leukemia detection: Exploring the impact of nesterov-accelerated adaptive moment estimation optimizer. *Biomed. Signal Process. Control* **94**, 106246. 10.1016/j.bspc.2024.106246 (2024).

[63] Hinton, G. E. & Salakhutdinov, R. R. Reducing the dimensionality of data with neural networks. *Science* **313**, 504–507 (2006). 16873662 10.1126/science.1127647

[64] Hassan, E. Enhancing coffee bean classification: A comparative analysis of pre-trained deep learning models. *Neural Comput. Appl.* **36**, 9023–9052. 10.1007/s00521-024-09623-z (2024).

[65] Zantye, M. S., Arora, A. & Hasan, M. F. Renewable-integrated flexible carbon capture: A synergistic path forward to clean energy future. *Energy Environ. Sci.* **14**, 3986–4008 (2021).

